# Anti-Inflammatory Polyketide Derivatives from the Sponge-Derived Fungus *Pestalotiopsis* sp. SWMU-WZ04-2

**DOI:** 10.3390/md20110711

**Published:** 2022-11-13

**Authors:** Peng Jiang, Jinfeng Luo, Yao Jiang, Liping Zhang, Liyuan Jiang, Baorui Teng, Hong Niu, Dan Zhang, Hui Lei

**Affiliations:** 1 School of Pharmacy, Southwest Medical University, Luzhou 646000, China; 2 Key Laboratory of Tropical Marine Bioresources and Ecology, Guangdong Key Laboratory of Marine Materia Medica, South China Sea Institute of Oceanology, Chinese Academy of Sciences, Guangzhou 510301, China; 3 University of Chinese Academy of Sciences, 19 Yuquan Road, Beijing 100049, China

**Keywords:** *Pestalotiopsis* sp., polyketide, sponge-derived fungus, anti-inflammatory activity

## Abstract

Five undescribed polyketide derivatives, pestaloketides A–E (**1**–**5**), along with eleven known analogues (**6**–**16**), were isolated from the sponge-derived fungus *Pestalotiopsis* sp. Their structures, including absolute configurations, were elucidated by analyses of NMR spectroscopic HRESIMS data and electronic circular dichroism (ECD) calculations. Compounds **5**, **6**, **9**, and **14** exhibited weak cytotoxicities against four human cancer cell lines, with IC_50_ values ranging from 22.1 to 100 μM. Pestaloketide A (**1**) is an unusual polyketide, featuring a rare 5/10/5-fused ring system. Pestaloketides A (**1**) and B (**2**) exhibited moderately inhibited LPS-induced NO production activity, with IC_50_ values of 23.6 and 14.5 μM, respectively, without cytotoxicity observed. Preliminary bioactivity evaluations and molecular docking analysis indicated that pestaloketides A (**1**) and B (**2**) had the potential to be developed into anti-inflammatory activity drug leads.

## 1. Introduction

Marine sponge-derived fungi have been proven to be a large and promising source of novel drug leads [1]. Among them, the *pestalotiopsis* species isolated from specific habitats are especially recognized as important producers of structurally varied, biologically active metabolites [2,3,4]. Since the discovery of taxol from the fungal *Pestalotiopsis microspora* [5], many novel secondary metabolites with potential pharmaceutical properties have been reported from this genus, including anti-inflammatory, cytotoxic, antiviral, antioxidant, and antimicrobial activities [6,7,8]. Accordingly, these findings have inspired many researchers to investigate the bioactive metabolites produced by *Pestalotiopsis* species. 

The *pestalotiopsis* species, mainly distributed in both terrestrial and marine habitats, can produce many secondary metabolites. Polyketides possessing a rearranged or a modified different carbon nucleus have been reported from *Pestalotiopsis* species [9,10]. However, a novel tricyclic 5/10/5 skeleton has not been declared. 

**Figure 1:**
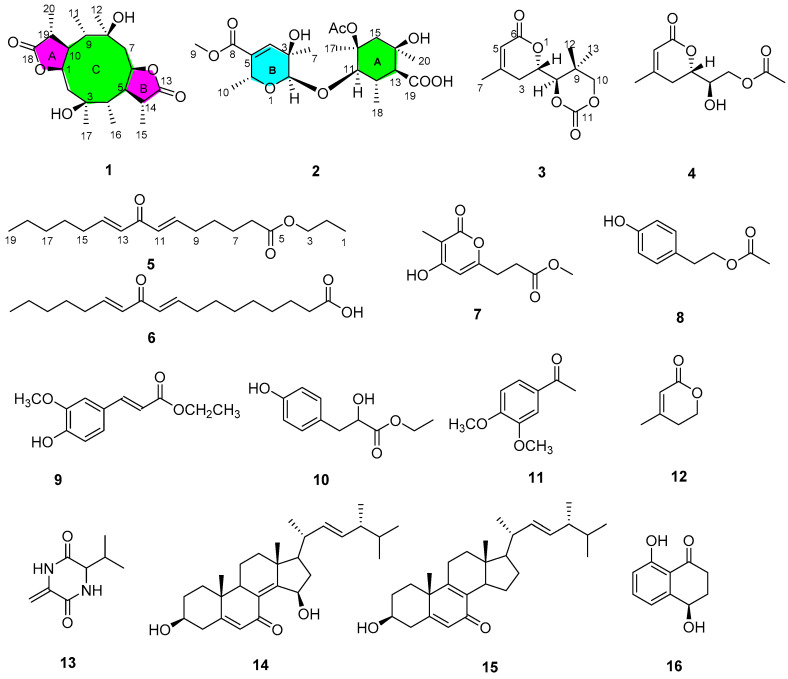
Structures of compounds **1–16**.

As part of our ongoing excavation for new secondary metabolites from sponge-derived fungi [11], the fungus *Pestalotiopsis* sp. was investigated by the “epigenetic modification” strategy, including 5-aza-2-deoxycytidine [12]. Chemical exploration of ethyl acetate (EtOAc) extract of the fungus led to the isolation of five new polyketide derivatives (**1**–**5**) (Figure 1), along with eleven known compounds (**6**–**16**). Herein, details of the isolation, structural elucidation, and bioactivities of the isolated compounds are described.

## 2. Results and Discussion

**Figure 2:**
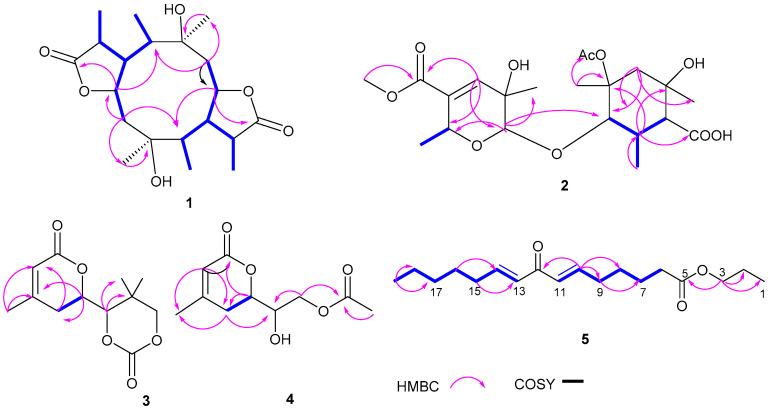
COSY and key HMBC correlations of **1–5**.

**Figure 3:**
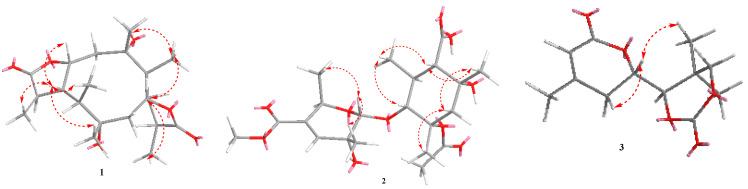
Key NOESY correlations of compounds **1**–**3**.

**Figure 4:**
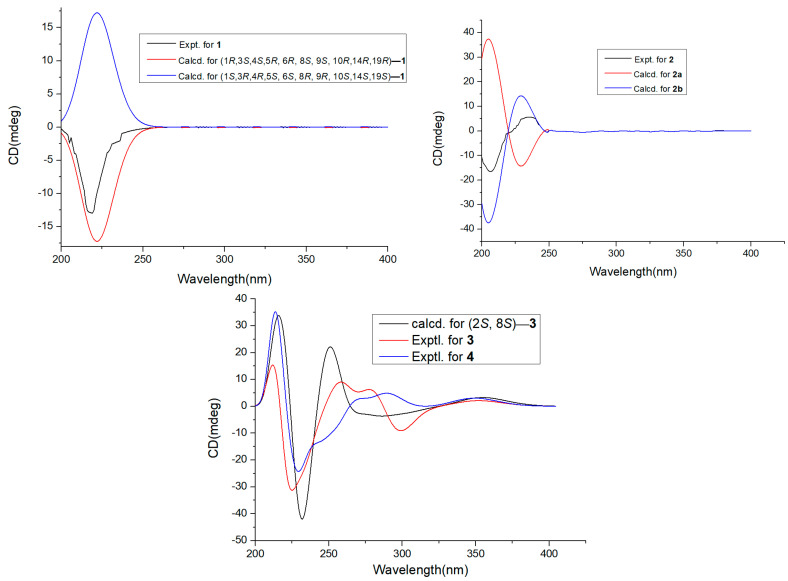
ECD spectra of **1**–**3** as well as experimental ECD spectrum **4**.

Compound **1** was detected as white oil. Its ^1^ H and ^13^C NMR data and HRESIMS spectrum data at *m*/*z* 391.2101 [M+Na]^+^ suggested that **1** had the molecular formula C_20_H_32_O_6_. Analysis of ^1^H NMR and HSQC spectrum (Table 1) of **1** showed six methyl groups (*δ*_H_ 0.95, d (*J* = 7.2 Hz, H_3_-11), 1.20, s, H_3_-12, 1.32, d (*J* = 7.6 Hz, H_3_-15), 0.97, d (*J* = 7.0 Hz, H_3_-16), 1.19, s, H_3_-17, and 1.29, d (*J* = 7.4 Hz, H_3_-20)), two oxygenated methines (*δ*_H_ 4.84, td (*J* = 2.4, 6.8 Hz, H-1), 4.93, td (*J* = 2.5, 7.3 Hz, H-6)), and two methylenes (*δ*_H_ 2.16, m; *δ*_H_ 2.21, m, 2.02, m). The ^13^C NMR and HSQC spectra of **1** exhibited 20 carbon signals, including six methyls, eight methines, two methylenes, and four oxygenated quaternary carbons. The above NMR data revealed the structure of **1** as a polyketide derivative, which was supported again by key HMBC correlations from H-19 (*δ*_H_ 2.88) to C-18 (*δ*_C_ 179.6), C-20 (*δ*_C_ 11.6), C-10 (*δ*_C_ 49.5), and C-9 (*δ*_C_ 44.0); and from H-14 (*δ*_H_ 2.70) to C-13 (*δ*_C_ 181.1), C-6 (*δ*_C_ 81.4), C-5 (*δ*_C_ 54.0), C-4 (*δ*_C_ 50.6), and C-15 (*δ*_C_ 18.3). The key HMBC correlations from H-2 (*δ*_H_ 2.16) to C-1 (*δ*_C_ 81.0), C-4 (*δ*_C_ 50.6), and C-17 (*δ*_C_ 23.8); and from H-7 (*δ*_H_ 2.02/2.21) to C-9 (*δ*_C_ 44.0), C-5 (*δ*_C_ 54.0), C-6 (*δ*_C_ 81.4), and C-12 (*δ*_C_ 23.8), the COSY correlations H-20/H_3_-19/H-10/H-9/H_3_-11/H-1/H-2 (fragment **Ⅰ**) and H_3_-16/H-4/H-5/H-14/H_3_-15/H-6/H-7 (fragment **Ⅱ**) (Figure 2) indicated that lactone rings (A and B) were connected by C-1 to C-10, C-5 to C-6 bond, which established a novel tricyclic 5/10/5 skeleton. The relative configuration of **1** was deduced from the key NOESY correlations (Figure 3). The cross-peaks from H-10 to H_3_-20, H_3_-11, and H-1; from H_3_-12 to H_3_-10 and H-6; and from H-5 to H_3_-15, H_3_-16, and H_3_-17, together with the correlation from H-1 to H-17, indicated that H-1, H-17, H_3_-16, H_3_-15, H-5, H-6, H_3_-12, H_3_-11, H_3_-20, and H-10 were on the same side. In order to discriminate between (1*S*, 3*R*, 4*R*, 5*S*, 6*S*, 8*R*, 9*R*, 10*S*, 14*S*, 19*S*)-**1** and (1*R*, 3*S*, 4*S*, 5*R*, 6*R*, 8*S*, 9*S*, 10*R*, 14*R*, 19*R*)-**1**, calculations of the ECD spectrum of **1** were performed. As a result, the calculated spectrum of (1*R*, 3*S*, 4*S*, 5*R*, 6*R*, 8*S*, 9*S*, 10*R*, 14*R*, 19*R*)-**1** coincided well with its experimental data (Figure 4), suggesting the absolute configuration of **1** to be 1*R*, 3*S*, 4*S*, 5*R*, 6*R*, 8*S*, 9*S*, 10*R*, 14*R*, 19*R.*

Compound **2** was yielded as a white solid. Its molecular formula C_21_H_32_O_10_ was established by ^13^C NMR data together with HRESIMS at *m*/*z* 443.1923, [M-H]^-^. Analysis of NMR spectra of **2** showed six methyl protons (*δ*_H_ 0.90, 1.04, 1.14, 1.30, 1.81, 1.35), one methylene protons (*δ*_H_ 2.12, 1.86), and one olefinic protons at *δ*_H_ 6.53 (1H, d, *J* = 2.0 Hz). The DEPT and ^13^C NMR spectra revealed 21 resonances including six methyl (*δ*_C_ 25.9, 18.0, 21.0, 14.2, 22.1, 22.6), one methylene (*δ*_C_ 45.7), six methines (*δ*_C_ 94.0, 139.5, 66.0, 80.2, 56.0, 43.9), seven nonprotonated carbons, one carbonyl group (*δ*_C_ 180.4), and two ester carbonyl groups (*δ*_C_ 179.5, 165.9). The key HMBC correlations from H-12 (*δ*_H_ 2.26) to C-13 (*δ*_C_ 43.9), C-18 (*δ*_C_ 14.2), C-11 (*δ*_C_ 80.2), C-16 (*δ*_C_ 74.4), and C-19 (*δ*_C_ 180.4); H-15 (*δ*_H_ 2.12, 1.85) to C-20 (*δ*_C_ 22.1), C-13 (*δ*_C_ 43.9), and C-14 (*δ*_C_ 80.0); H-17 (*δ*_H_ 1.35) to C-16 (*δ*_C_ 74.4), and C-1′ (*δ*_C_ 179.5), together with ^1^H-^1^H COSY correlation information between H-18/H-12/H-13/H-11 established the fragment A (Figure 2). Further, ^1^H-^1^H COSY correlation information between H-10 (*δ*_H_ 1.30) and H-6 (*δ*_H_ 4.54), along with the key HMBC correlations from H-4 (*δ*_H_ 6.53) to C-8 (*δ*_C_ 165.9), C-5 (*δ*_C_ 133.7), C-2 (*δ*_C_ 94.1), C-7 (*δ*_C_ 21.0), and C-6 (*δ*_C_ 66.0); and H-2 (*δ*_H_ 4.80) to C-4 (*δ*_C_ 139.5), C-6 (*δ*_C_ 66.0), C-7 (*δ*_C_ 21.0) and C-11 (*δ*_C_ 80.2) established the fragment B. Therefore, the structure of **2** was assigned (Figure 1). The relative configurations of **2** were investigated by key NOESY spectrum, as indicated in Figure 3. The key NOESY correlations of H-2/H_3_-10, H-2/H_3_-7, H_3_-20/H_3_-18, H_3_-20/H_3_-17, H-13/H_3_-18, and H-11/H_3_-17 in **2**, suggested these groups were cofacial. In order to confirm the absolute configurations of **2**, the ECD calculations were performed (**2a** and **2b**) (Figure 4). As a result, the ECD calculations of **2b** fitted well with the experimental curve. Thus, compound **2** was assigned and named as pestaloketide B.

Compound **3** was detected as yellow oil, giving the molecular formula of C_12_H_16_O_5_ from the analysis of their ^13^C NMR data and HRESIMS (Table 2). Carefully, analysis of the 1D NMR data, in combination with the HSQC spectrum, revealed characteristic signals corresponding to one olefinic proton (*δ*_H_ 5.83 (1H, s, H-5)) and three methyls (*δ*_H_ 1.08, s, *δ*_H_ 1.24, s, *δ*_H_ 2.03, s). The ^13^C NMR and HSQC spectroscopic data of **3** exhibited resonances for one lactone carbon (*δ*_C_ 164.8 (C-6)) and three oxygenated carbons (*δ*_C_ 65.9, 75.7, 76.4). Detailed analysis of these above data of **3** revealed that compound **3** was very similar to those of **12**, 3-methyl-2-penten-5 [13], except for the presence of the lactonic ring groups at C-2 in **3.** The aforementioned conclusion was supported again by the key correlations from H-7 to C-3/C-5/C-4, from H-8 to C-9/C-12/C-13, and from H-2 to C-3/C-4/C-9/C-6. The key NOESY correlations of H-2 with H_3_-12 and H-3*β* suggested that these protons were cofacial; thus, the relative configuration of compound **3** was deduced as 2*S*, 8*S*. The absolute configuration of C-2 and C-8 in **3** were elucidated by comparing the calculated ECD spectrum of the 2*S*, 8*S*-model and the experimental ECD curve of **3** (Figure 4). Thus, compound **3** was assigned (Figure 1).

Compound **4** was yielded as a yellow oil, gave the molecular formula of C_10_H_14_O_5_ from the HRESIMS ion at *m*/*z* 237.0747 [M+Na]^+^ and ^13^C NMR data. Analysis of the 1D NMR spectroscopic data between **4** and **3** indicated both compounds to be structurally similar (Table 2). The major difference was that the lactonic ring groups at C-2 in **3** were replaced by one acetate at C-2 in **4**. The aforementioned results were supported by the key HMBC correlations from H-5 to C-6/C-3/C-7 (*δ*_C_ 22.9), from H-2 to C-3/C-4/C-6, and from H-3 to C-7/ C-2/C-5/C-8, and the COSY correlations H-2 and H-3. According to the above evidence, by the biosynthetic pathway, similar chemical shift, and specific rotation (**4**, [α${]}_{D}^{25}$-52 (*c* 0.2, MeOH, **3**[α${]}_{D}^{25}$-58 (*c* 0.20, MeOH)) data comparison, the relative configurations of **3** and **4** were concluded to be the same for C-2 and C-8. This assignment was proved by the ECD spectrum, the result of which showed good accordance with **3** (Figure 4). Thus, the structure of **4** was assigned and named pestaloketide C.

Compound **5** was a colorless oil. The ^1^H NMR and HSQC data revealed the presence for four olefinic methines (*δ*_H_ 6.97 (1H, dt, *J* = 15.6, 7.0 Hz), 6.94 (1H, dt, *J* = 15.6, 7.0 Hz), 6.4 (2H, d, *J* = 15.6 Hz)), eleven aliphatic methylenes (*δ*_H_ 1.34-2.26), and two methyls (*δ*_H_ 0.92 (3H, t, *J* = 6.0 Hz); *δ*_H_ 0.99 (3H, d, *J* = 6.7 Hz)). The ^13^C NMR and HSQC data (Table 2) of **5** showed 18 carbon resonances comprising two methyls, four olefinic methines, ten aliphatic methylenes, one oxygenated carbon, and two carbonyl carbons. The aforementioned data suggested that **5** was similar to compound **6**, 11-keto-9(*E*), 12(*E*)-octadecadienoic acid [14]. The major difference was the absence of the carboxyl group and the presence of a butyl ester (*δ*_C_ 169.3 72.9, 29.1, 19.5) in **6**. HMBC correlations from H-10 to C-12/C-11/C-9/C-8, from H-9 to C-12/C-10/C-9, and from H-3 to C-5/C-2/C-1, along with ^1^H-^1^H COSY correlations of H-13/H14/H-15/H-16/H17/H-18/H-19 and H-11/H10/H-9/H-8/H7/H-6 confirmed the planar structure of **5**.

In order to further investigate the structure of compound **5**, ESI-MS analysis was performed. It was found that compound **5** yielded the ion at *m*/*z* 294 in the mass spectrum, and further fragmentation of this ion gave an intense signal at *m*/*z* 179 [Frag 1] in Figure S35. Finally, the results of the ESI-MS analysis suggested that fragment at *m*/*z* 179 was attributed to [CH_3_(CH_2_)_4_(CH)_2_CO(CH)_2_(CH_2_)_2_] cations. These results assisted to reconfirm the configuration of compound **5**.

Compounds **6**–**16** were determined to be the known 11-keto-9(*E*),12(*E*)-octadecadienoic acid (**6**) [14], scirpyrone K (**7**) [15], 4-hydroxy phenethyl acetate (**8**) [16], ethyl (2*E*)-3-(4-hydroxy-3-methoxyphenyl)prop-2-enoate (**9**) [17], ethylp-hydroxyphenyllactate (**10**) [18], 3,4-dimethoxyacetophenone (**11**) [19], 4-methyl-5,6-dihydropyren-2-one (**12**) [20], cyclo(L-Val-Dha) (**13**) [21], 3,15-dihydroxyl-(22*E*, 24 *R*)-ergosta-5,8(14),22-trien-7-one (**14**) [22], 3*β*-hydroxy-(22*E*,24*R*)-ergosta-5,8,22-trien-7-one (**15**) [23], and (-)-isosclerone (**16**) [24], by comparing their NMR data. 

The cytotoxicity assay of compounds (**1**–**16**) was examined via MTT assay. Compounds **5**, **6**, **9**, and **14** showed weak cytotoxicities against four human cancer cell lines, with IC_50_ values 22.1-100 μM (Table 3). Anti-inflammatory activities were performed for compounds **1**–**4**, **7**–**8**, **10**–**13**, and **15**–**16** with NO production inhibitory activity. Pestalolactones A (**1**) and B (**2**) showed moderate inhibitory of NO production with IC_50_ values of 23.6 and 14.5 μM, respectively, without cytotoxicity observed. Others were inactive (100 μM). The result showed that pestalolactones A (**1**) and B (**2**) had the potential to be developed into anti-inflammatory activity drug leads (Table 4).

**Figure 5:**
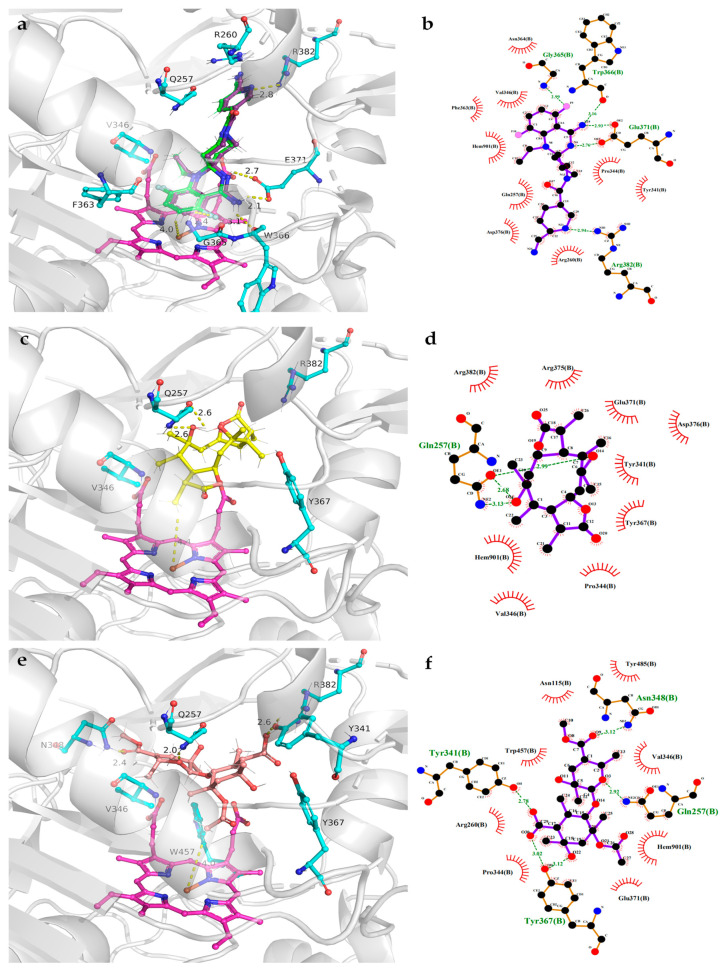
Representative docking poses of dexamethasone and pestaloketides A (**1**) and B (**2**) bound to the INOS protein (PDB ID:3E6T). Dexamethasone was used for redocking. The intermolecular interactions between INOS with dexamethasone and pestaloketides A (**1**) and B (**2**) are depicted as three-dimensional interaction maps ((**a**,**c**,**e**), respectively) and two-dimensional interaction maps ((**b**,**d**,**f**), respectively).

Interestingly, pestaloketide A (**1**) is reported to be the first tricyclic 5/10/5 skeleton polyketide from the sponge-derived fungus *Pestalotiopsis* sp. In addition, pestaloketides A (**1**) and B (**2**) exhibited moderately inhibited LPS-induced NO production activity. To further investigate the anti-inflammatory mechanism of pestaloketides A (**1**) and B (**2**), molecular docking of **1** and **2** with inducible NO oxidase (iNOS) as target was employed, and dexamethasone was used for redocking (Figure 5). Docking results display that the docking pose of dexamethasone (Figure 5a, green) fit well with its original pose (Figure 5a, purple) in cocrystal, and compounds **1**–**2** exhibited good interactions with the INOS target in its pocket. Pestaloketide A (**1**) had a hydrogen bond interaction with Q257, and had nonpolar interactions with residues V346, Y367, and R382 and the cofactor heme. For pestaloketide B (**2**), hydrogen bond interactions were formed with residues Q257, Y341, N348 and Y367, and nonpolar interactions were formed with residues V346, R382, and W457 and the cofactor heme. These results indicate that **2** has a stronger association with the INOS protein than **1,** which is consistent with our in vitro biological activity experiment results. Therefore, both pestalolactones A (**1**) and B (**2**) had the potential to be developed into anti-inflammatory activity drug leads.

## 3. Materials and Methods

### 3.1. General Experimental Procedures

Optical rotations were measured using an (Anton) MCP500 polarimeter. HRESIMS were used for a Bruker maXis TOF-Q mass spectrometer, while infrared spectra were acquired on a Shimadzu IR spectrometer. NMR spectra were measured on Bruker spectrometer. The UV spectra were carried out on a Shimadzu UV-2600 PC spectrometer. Open chromatography column was performed on silica gel (100-300 mesh, Qingdao, China), YMC ODS-A (S-50 μm, 12 nm) (YMC Co., Ltd., Kyoto, Japan). HPLC was accomplished using ODS column (YMC-5μm, ODS-A) Sephadex LH-20 (GE, Sweden). The RAW 264.7 cells were obtained from the Chinese Academy of Sciences (Shanghai, China)

### 3.2. Fungal Material

Strain SWMU-WZ04-2 was obtained from the sponge collected in Weizhou Island, Guangxi province, China, in April 2018. It was identified as Pestalotiopsis sp. SWMU-WZ04-2 according to a molecular biological protocol by DNA amplification and sequencing of the ITS region. A voucher specimen (No. SWMU-WZ04-2) was deposited in the laboratory.

### 3.3. Fermentation, Extraction, and Isolation

The mass fermentation of the fungal strain SWMU-WZ04-2 was performed in 120 × 1 L Erlenmeyer flasks. The medium was grown (containing 200 g of natural rice, 3% sea salt; 200 mL of water, 10 μM of 5-aza-2-deoxycytidine) for 36 days at 28 °C. The fermented rice cultures were soaked and extracted with EtOAc to gain 59 g of residue.

The crude extract was chromatographed by silica gel cc (column chromatography), which was eluted with petroleum ether/EtOAc (50: 1 to 0: 1, *v*/*v*) and separated into 8 fractions (Fr-1–Fr-8). Fr-3 was applied to silica gel cc (petroleum ether/EtOAc, 10:1-5:1) to obtain five subfractions (Frs. 3.1-3.5). Fr. 3.3 was purified with Sephadex LH-20 (MeOH) and applied by C_18_ HPLC (80% H_2_O/MeOH) to obtain compounds 8 (6.0 mg) and **10** (4.0 mg). Fr. 3.4 was applied by ODS column chromatography eluting with MeOH/H_2_O (60%) and purified by Sephadex LH-20 column (MeOH) and HPLC (70%, MeOH/H_2_O) to give **9** (5.0 mg) and **11** (3.0 mg). Fr-4 was subjected by silica gel (petroleum ether-EtOAc, 6:1-0:1) and further separated by preparative TLC, (60%, H_2_O/MeOH), HPLC, and Sephadex LH-20 (MeOH), to yield **1** (7.0 mg), **3** (3.0 mg), and **4** (4.0 mg). Fr-5 was applied to silica gel cc (CH_2_Cl_2_/Acetone), followed by Sephadex LH-20 chromatography (MeOH) and HPLC (55% H_2_O/MeOH) to afford **2** (4.0 mg). Fr. 5.4 was applied by ODS (H_2_O/MeOH) column and further subjected by HPLC (H_2_O/MeOH 45:55) to obtain **7** (7.0 mg), **12** (3.0 mg), and **16** (5.0 mg). Fr-6 was divided into five subfractions (Frs. 6.1-6.5) by silica gel cc (CH_2_Cl_2_-MeOH,10:1- 0:1). Then, Fr. 6.3 was applied by HPLC (MeOH/H_2_O, 45%) to yield **5** (4.0 mg). Fr. 6.4 was applied by HPLC (MeOH/H_2_O, 40%) to afford **6** (8.0 mg). Fr. 6.5 was applied by Sephadex LH-20 (MeOH), preparative TLC and HPLC (H_2_O/MeOH) to yield **13** (6.0 mg), **14** (12.0 mg), and **15** (10.0 mg).

*Pestaloketide A* (**1**): white oil; [α${]}_{D}^{25}$−31 (c 0.45, MeOH); UV (MeOH) λmax(log *ε*) 210 (2.54) nm; IR (film)*ν*max 3329, 2952, 2851, 1680, 1589, 1440, 1348, 1024 cm^−1^; ^1^H NMR and ^13^C NMR data, Table 1; HRESIMS *m*/*z* 391.2101 [M+Na]^+^ (calcd for C_20_H_32_NaO_6_, 391.2109).

*Pestaloketide B* (**2**): white powder; [α${]}_{D}^{25}$+42 (c 0.1, MeOH); UV (MeOH) *λ*max(log *ε*) 205 (3.60), 233 (3.32) nm; IR (film)*ν*max 3368, 2925, 2859, 1702, 1646, 1604, 1380, 1349, 1258, 1226, 1121, 1053, 1034, 878 cm^−1^; ^1^H NMR and ^13^C NMR data, Table 1; HRESIMS at *m*/*z* 443.1923 [M-H]^−^ (calcd for C_21_H_31_O_10_, 443.1924).

*Pestaloketide C* (**3**): yellow oil; [α${]}_{D}^{25}$−58 (c 0.2, MeOH); UV (MeOH) *λ*max(log *ε*) 235 (4.26), 210 (3.38) nm; IR (film)*ν*max 3329, 2952, 2852, 1732, 1680, 1467, 1440, 1379, 1220, 1165, 1103 cm^−1^; ^1^H NMR and ^13^C NMR data, Table 2; HRESIMS *m*/*z* 263.1005 [M+Na]^+^ (calcd for C_12_H_16_NaO_5_, 263.0902).

*Pestaloketide D* (**4**): yellow oil; [α${]}_{D}^{25}$−52 (c 0.2, MeOH); UV (MeOH) *λ*max(log *ε*) 240 (4.23), 205 (3.26) nm; IR (film)*ν*max 3359, 3262, 2937, 1705, 1652, 1580, 1455, 1376, 1349, 1166, 1022 cm^−1^; ^1^H NMR and ^13^C NMR data, Table 2; HRESIMS *m*/*z* 237.0747 [M+Na]^+^ (calcd for C_10_H_14_NaO_5_, 237.0725).

*Pestaloketide E* (**5**): colorless oil; [α${]}_{D}^{25}$−26 (c 0.1, MeOH); UV (MeOH) *λ*max(log *ε*) 250 (4.16) nm; IR (film)*ν*max 2930, 2853, 1705, 1662, 1455, 1348, 1162, 950, 836, 710, 680 cm^−1^; ^1^H NMR (600 Hz) and ^13^C NMR(150 Hz) data, Table 2; HRESIMS *m*/*z* 293.2128 [M-H]^-^ (calcd for C_18_H_29_O_3_, 293.2126).

### 3.4. Computational Section

The calculations were applied by the Spartan’14, Gaussian 09 software, and Merck Molecular Force Field (MMFF), respectively. The conformers of **1**–**4** were chosen at the B3LYP/6-311+G(d,p) level. The overall calculation of the ECD was performed using the TDDFT method for the stable conformers of new compounds. The spectra were obtained by SpecDis 1.6.

### 3.5. Cytotoxicity Assay

The method for the assay of cytotoxicity activity of **1**–**16** was conducted according to the one described previously [11]. Positive control (Adriamycin).

### 3.6. Inhibition of NO Production Assays

The activity of compounds **1**–**16** were examined by inhibited NO production in LPS-stimulated RAW. The detailed process of the assay is described in the previously published paper [9]. Positive control (dexamethasone).

### 3.7. Molecular Docking

The three-dimensional structure of INOS (PDB ID:3E6T) was acquired from the Protein Data Bank (http://www.rcsb.org, accessed on 30 October 2022) [25,26], for which the resolution was 2.5 Å. Using the Chain A of the INOS structure as the receptor, pestaloketides A (**1**) and B (**2**) were docked using Autodock vina [27] and AutoDockTools-1.5.6 [28]. The geometrical restraints for **1** and **2** were generated by Grade Web Server (http://grade.globalphasing.org, accessed on 29 October 2022). A grid box of a 48.02 Å × 42.58 Å × 33.75 Å size was centered on the catalytic site. All docking parameters were set to default values. The docking results were further analyzed and presented using PyMOL (http://www.pymol.org, accessed on 29 October 2022) and LigPlot^+^ [29].

## 4. Conclusions

In summary, five new compounds (**1**–**5**), together with other eleven known natural products (**6**–**16**), were isolated from the fungus *Pestalotiopsis* sp. SWMU-WZ04-2. Pestaloketide A (**1**) is an unusual polyketide featuring a rare 5/10/5-fused ring system. Compounds **5**, **6**, **9**, and **14** showed weak cytotoxicities against four human cancer cell lines (IC_50_: 22.1–100 μM). Other compounds were inactive (100 μM). Anti-inflammatory activities were performed for compounds **1**–**4**, **7**–**8**, **10**–**13**, and **15**–**16**, and Pestalolactones A (**1**) and B (**2**) showed moderate inhibitory of NO production with IC_50_ values of 23.6 and 14.5 μM, respectively, without cytotoxicity observed.” Although the detailed mechanism of action is still undefined for pestaloketides A (**1**) and B (**2**), molecular docking analysis showed that both compounds had the potential to be developed into anti-inflammatory activity drug leads.

## Figures and Tables

**Table 1:** ^1^ H (NMR) (500 MHz) and ^13^C NMR (125 MHz) data for 1 and 2.

	1 ^a^		2 ^b^	
No.	dC, Type	dH (J in Hz)	dC, Type	dH (J in Hz)
1	81.0, CH	4.84, td (2.4, 6.8)	-	-
2	46.0, CH_2_	2.16, m	94.1, CH	4.80, s
3	81.2, C	-	67.2, C	-
4	50.6, CH	1.94, dt (7.0, 13.1)	139.5, CH	6.53, d (2.0)
5	54.0, CH	2.14, m	133.7, C	-
6	81.4, CH	4.93, td (2.5, 7.3)	66.0, CH	4.54, dd (6.7, 1.6)
7	46.4, CH_2_	2.21, m, 2.02, m	21.0, CH_3_	1.14, s
8	80.9, C	-	165.9, C	-
9	44.0, CH	2.04, m	50.8, OCH_3_	3.65, s
10	49.5, CH	2.54, dt (10.0, 7.1)	18.0, CH_3_	1.30, d (6.7)
11	15.9, CH_3_	0.95, d (7.2)	80.2, CH	4.78, s
12	23.8, CH_3_	1.20, s	56.0, CH	2.26, d (7.2)
13	181.1, C	-	43.9, CH	2.09, m
14	42.6, CH	2.70, dd (7.6, 3.2)	80.0, C	-
15	18.3, CH_3_	1.32, d (7.6)	45.7, CH_2_	2.12, m, 1.85, m
16	16.0, CH_3_	0.97, d (7.0)	74.4, C	-
17	23.8, CH_3_	1.19, s	25.9, CH_3_	1.35, s
18	179.6, C	-	14.2, CH_3_	0.90, d (7.2)
19	38.3, CH	2.88, dq (10.0, 7.4)	180.4, C	-
20	11.6, CH_3_	1.29, d (7.4)	22.1, CH_3_	1.04, s
1′-OAc			179.5, C	-
2′			22.6, CH_3_	1.81, s

^a^ Measured in CDCl_3._^b^ Measured in CD_3_OD.

**Table 2:** ^1^ H (NMR) (500 MHz) and ^13^C NMR (125 MHz) data for **3**–5.

	3 ^a^		4 ^b^		5 ^b^	
No.	dC, Type	dH (J in Hz)	dC, Type	dH (J in Hz)	dC, Type	dH (J in Hz)
1					19.5, CH_3_	0.99, d (6.7)
2	65.9, CH	4.38,t(6.3)	67.6, CH	4.38, t (6.3)	29.1, CH_2_	1.5, m
3	29.2, CH_2_	2.38, t (6.3)	29.3, CH_2_	2.44, t (6.3)	72.9, CH_2_	4.07, t (6.7)
4	157.8, C		162.1, C			
5	116.8, CH	5.83, s	116.5, CH	5.78, s	169.3, C	
6	164.8, C		167.5, C		30.7, CH_2_	1.34, m
7	23.0, CH_3_	2.03, s	22.9, C	2.02, s	29.4, CH_2_	1.5, m
8	75.7, CH	4.12, d (6.3)	68.3, CH	4.02, m	30.4, CH_2_	1.33, m
9	40.9, C		66.2, CH_2_	4.10, dd (11.3, 5.3)	33.7, CH_2_	2.26, t (7.0)
10	76.4, CH_2_	4.03, d (8.9), 3.95, d (8.9)			150.4, CH	6.97, dt (15.6, 7.0)
11	177.6, C		172.6, C		129.4, CH	6.4, d (15.6)
12	22.9, CH_3_	1.24, s	20.7, CH_3_	2.06, s	192.0, C	
13	18.8, CH_3_	1.08, s			129.4, CH	6.4, d (15.6)
14					150.4, CH	6.94, dt (15.6, 7.0)
15					33.7, CH_2_	2.26, t (7.0)
16					30.4, CH_2_	1.35, m
17					32.6, CH_2_	1.33, m
18					23.5, CH_3_	1.30, m
19					14.4, CH_3_	0.92, t (6.0)

^a^ Measured in CDCl_3._^b^ Measured in CD_3_OD.

**Table 3:** Cytotoxicity of compounds **1**–**16** ^a^ (IC_50_ in μM).

Compound	SMMC-7721	H460	PC-3	BGC-823
5	65.1	35.6	28.2	>100
6	57.3	42.6	22.4	>100
9	35.0	54.3	42.0	22.1
14	73.5	64.3	>100	62.6
Adriamycin	2.2	1.2	1.8	1.5

^a^ Compounds that are not shown in this table did not exhibit activity (>100).

**Table 4:** Anti-inflammatory activities of the compounds **1**–**4**, **7**–**8**, **10**–**13**, and **15**–**16** (IC_50_, μM).

Compound	1	2	3–4, 7–8, 10–13, 15–16	Positive ^a^
IC_50_	23.6	14.5	-	12.1

^a^ Dexamethasone, - not exhibit activity.

## Data Availability

The data presented in this study are available in the main text and the supplementary materials of this article.

## Supplementary Materials

The following supporting information can be downloaded at https://www.mdpi.com/article/10.3390/md20110711/s1, Figures S1–S35,S37,S38: 1D, 2D NMR, and HRESIMS spectra of compounds **1**–**5**. S36: Computational details for compound **1**–**4** (ECD). Table SI–S3: Energy analysis for conformers of **1–3** at B3LYP/6-31G(d) level.
