# Supplementary material for: Anti-Inflammatory Polyketide Derivatives from the Sponge-Derived Fungus Pestalotiopsis sp. SWMU-WZ04-2
Source: Mar Drugs. 2022 Nov 13;20(11):711. doi: 10.3390/md20110711 (PMC9697532; doi:10.3390/md20110711)
Supplement: Supplementary file 1 [file marinedrugs-20-00711-s001.zip › marinedrugs-1992808-supplementary.pdf]

Supporting Information

Anti-inflammatory Polyketide Derivatives from the Sponge-Derived Fungus *Pestalotiopsis* sp. SWMU-WZ04-2

Peng Jiang [1,2,3], Jinfeng Luo [1], Yao Jiang [1], Liping Zhang [2], Liyuan Jiang [1], Baorui Teng [1], Hong Niu [1], Dan Zhang [1,*], Hui Lei [1,*]

1. School of Pharmacy, Southwest Medical University, Luzhou 646000, China
2. Key Laboratory of Tropical Marine Bioresources and Ecology, Guangdong Key Laboratory of Marine Materia Medica, South China Sea Institute of Oceanology, Chinese Academy of Sciences, Guangzhou 510301, China
3. University of Chinese Academy of Sciences, 19 Yuquan Road, Beijing 100049, China

Corresponding author.

zhangdan@swmu.edu.cn (D.Z.); huilei@swmu.edu.cn (H.L.); Tel.: +86-0830-3162291 (H.L.)

Figure S1. HRESI-MS spectrum of the new compound **1**

Figure S2. ^{1}H NMR (500 MHz, $CDCl_3$) spectrum of the new compound **1**

Figure S3. ^{13}C NMR (125 MHz, $CDCl_3$) spectrum of the new compound **1**

Figure S4. HSQC spectrum of the new compound **1**

Figure S5. HMBC spectrum of the new compound **1**

Figure S6. COSY spectrum of the new compound **1**

Figure S7. NOESY spectrum of the new compound **1**

Figure S8. HRESI-MS spectrum of the new compound **2**

Figure S9. ^{1}H NMR (500 MHz, CD_3OD) spectrum of the new compound **2**

Figure S10. ^{13}C NMR (125 MHz, CD_3OD) spectrum of the new compound **2**

Figure S11. HSQC spectrum of the new compound **2**

Figure S12. HMBC spectrum of the new compound **2**

Figure S13. COSY spectrum of the new compound **2**

Figure S14. NOESY spectrum of the new compound **2**

Figure S15. HRESI-MS spectrum of the new compound **3**

Figure S16. ^{1}H NMR (500 MHz, $CDCl_3$) spectrum of the new compound **3**

Figure S17. ^{13}C NMR (125 MHz, $CDCl_3$) spectrum of the new compound **3**

Figure S18. HSQC spectrum of the new compound **3**

Figure S19. HMBC spectrum of the new compound **3**

Figure S20. COSY spectrum of the new compound **3**

Figure S21. NOESY spectrum of the new compound **3**

Figure S22. HRESI-MS spectrum of the new compound **4**

Figure S23. ^{1}H NMR (500 MHz, CD_3OD) spectrum of the new compound **4**

Figure S24. ^{13}C NMR (125 MHz, CD_3OD) spectrum of the new compound **4**

Figure S25. HSQC spectrum of the new compound **4**

Figure S26. HMBC spectrum of the new compound **4**

Figure S27. COSY spectrum of the new compound **4**

Figure S28. NOESY spectrum of the new compound **4**

Figure S29. HRESI-MS spectrum of the new compound **5**

Figure S30. ^{1}H NMR (500 MHz, CD_3OD) spectrum of the new compound **5**

Figure S31. ^{13}C NMR (125 MHz, CD_3OD) spectrum of the new compound **5**

Figure S32. HSQC spectrum of the new compound **5**

Figure S33. HMBC spectrum of the new compound **5**

Figure S34. COSY spectrum of the new compound **5**

Figure S35. ESI-MS spectrum of compound **5**.

S36.Computational details for compound **1-4** (ECD)

Figure S37. Comparison of the calculated ECD spectra for **1** with the experimental spectrum of **1**

Figure S38. Calculated and experimental ECD spectras of **1** using model.

Table S1. Energy analysis for conformers of **1** at B3LYP/6-31G(d) level

Table S2. Energy analysis for conformers of **2** at B3LYP/6-31G(d) level

Table S3. Energy analysis for conformers of **3** at B3LYP/6-31G(d) level

Qualitative Analysis Report

**Data Filename**	J-13-POS.d	**Sample Name**	Sample9
**Sample Type**	Sample	**Position**	P1-A9
**Instrument Name**	Instrument 1	**User Name**	
**Acq Method**	default-20200902-pos.m	**Acquired Time**	7/1/2021 9:47:15 AM
**IRM Calibration Status**		**DA Method**	analysis.m
**Comment**			

Sample Group **Info.**

User Spectra

Fragmentor Voltage	Collision Energy	Ionization Mode
75	0	ESI

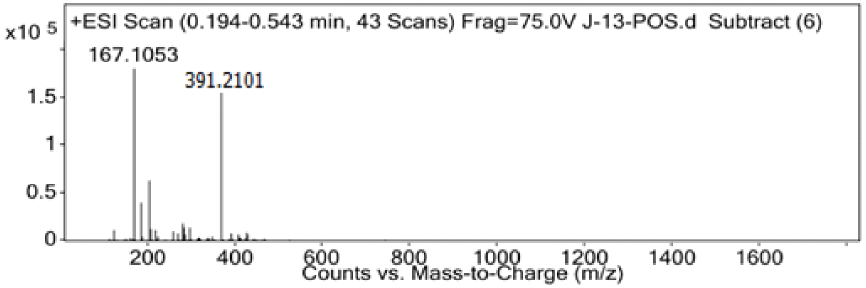

Figure S1. HRESI-MS spectrum of the new compound **1**

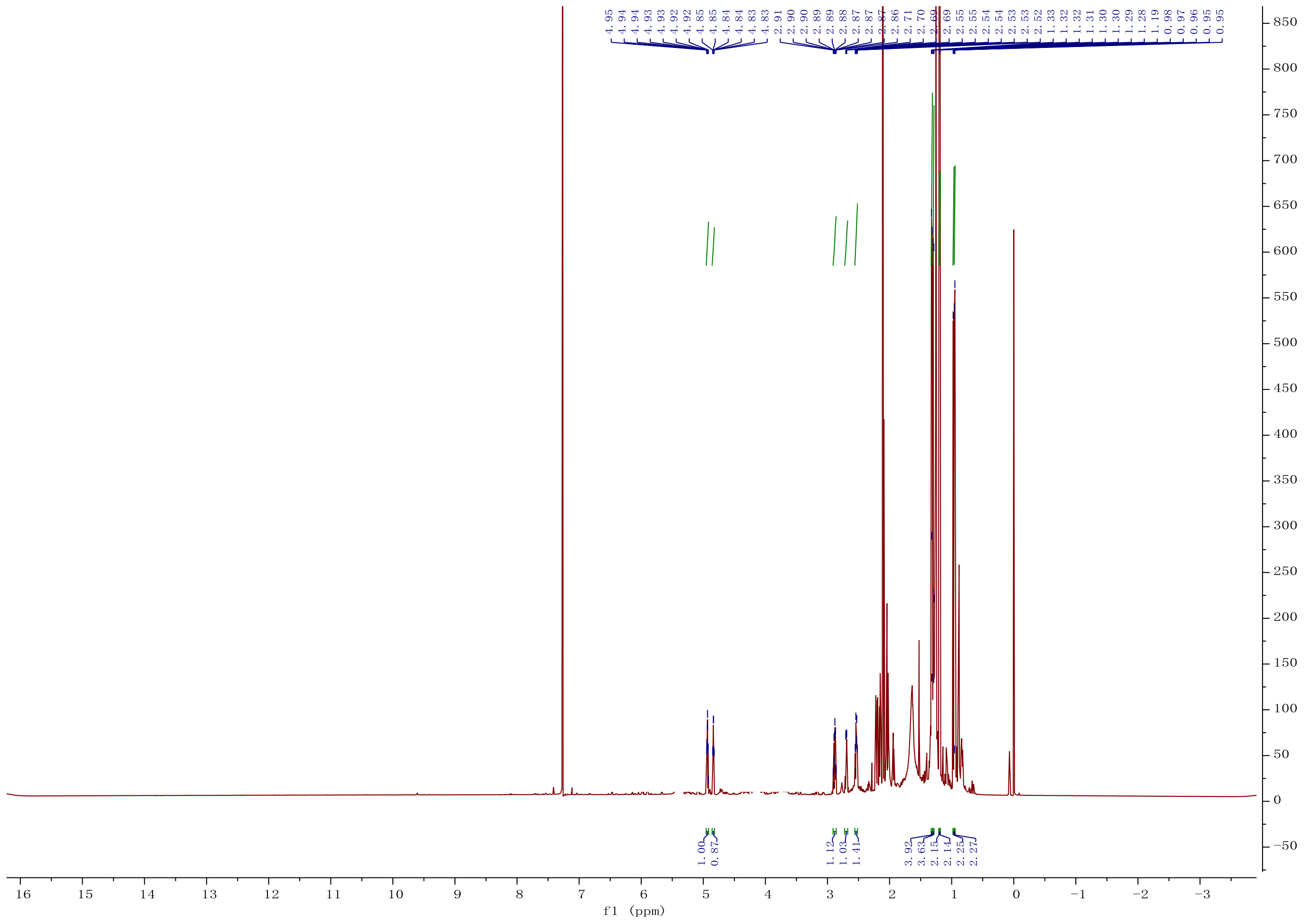

Figure S2. ^{1}H NMR (600 MHz, $CDCl_3$) spectrum of the new compound **1**

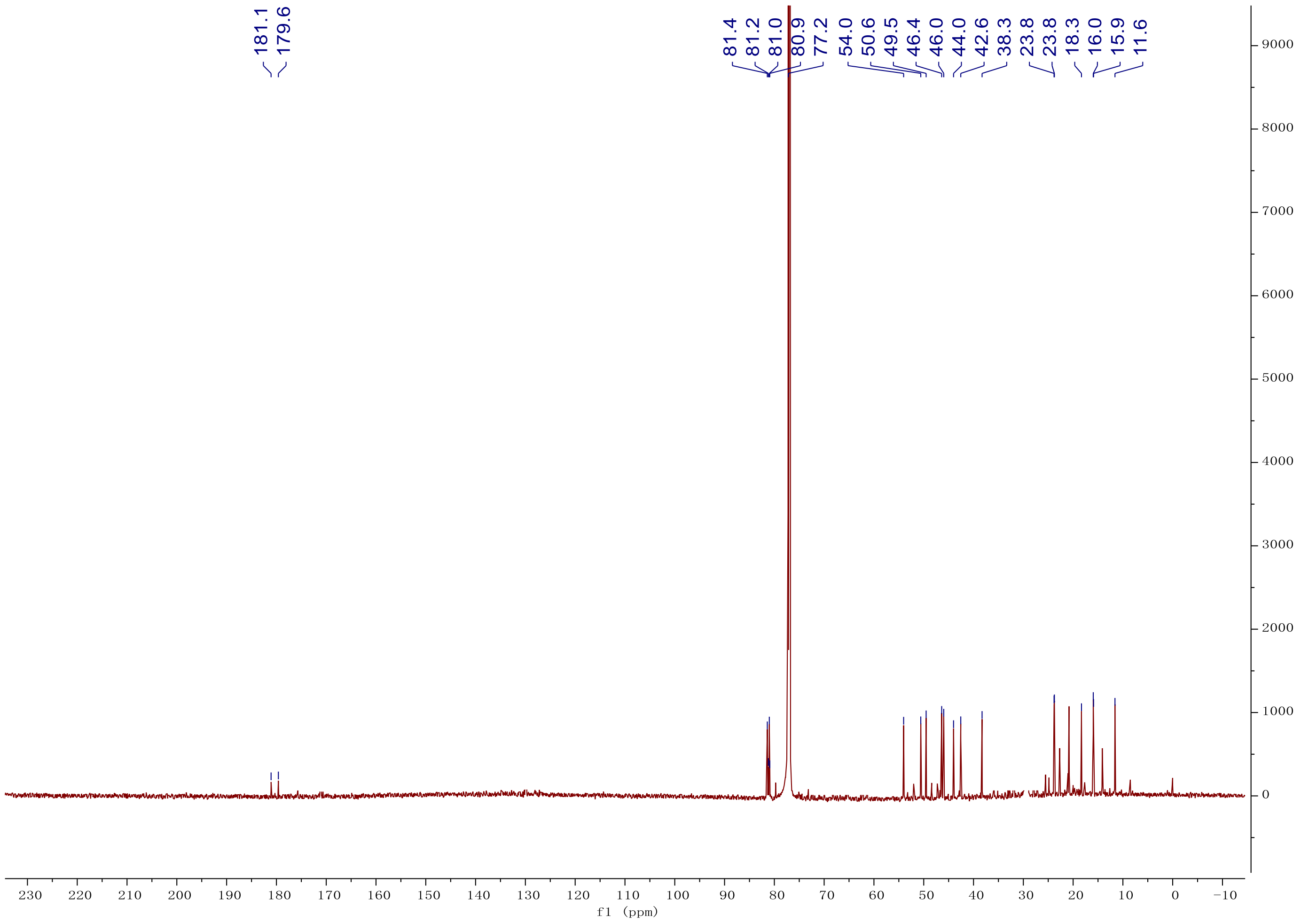

Figure S3. ^{13}C NMR (150 MHz, $CDCl_3$) spectrum of the new compound **1**

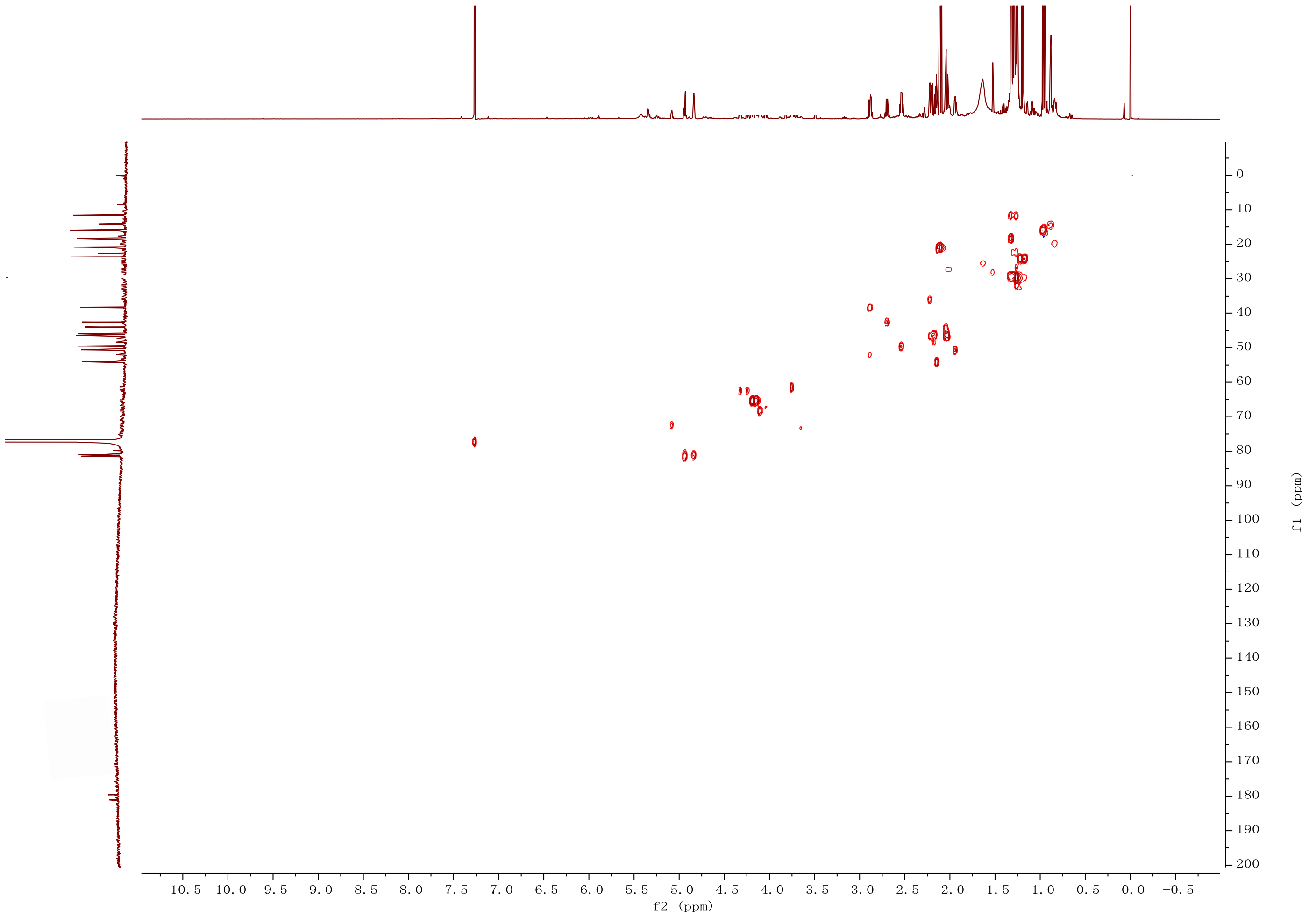

Figure S4. HSQC spectrum of the new compound **1**

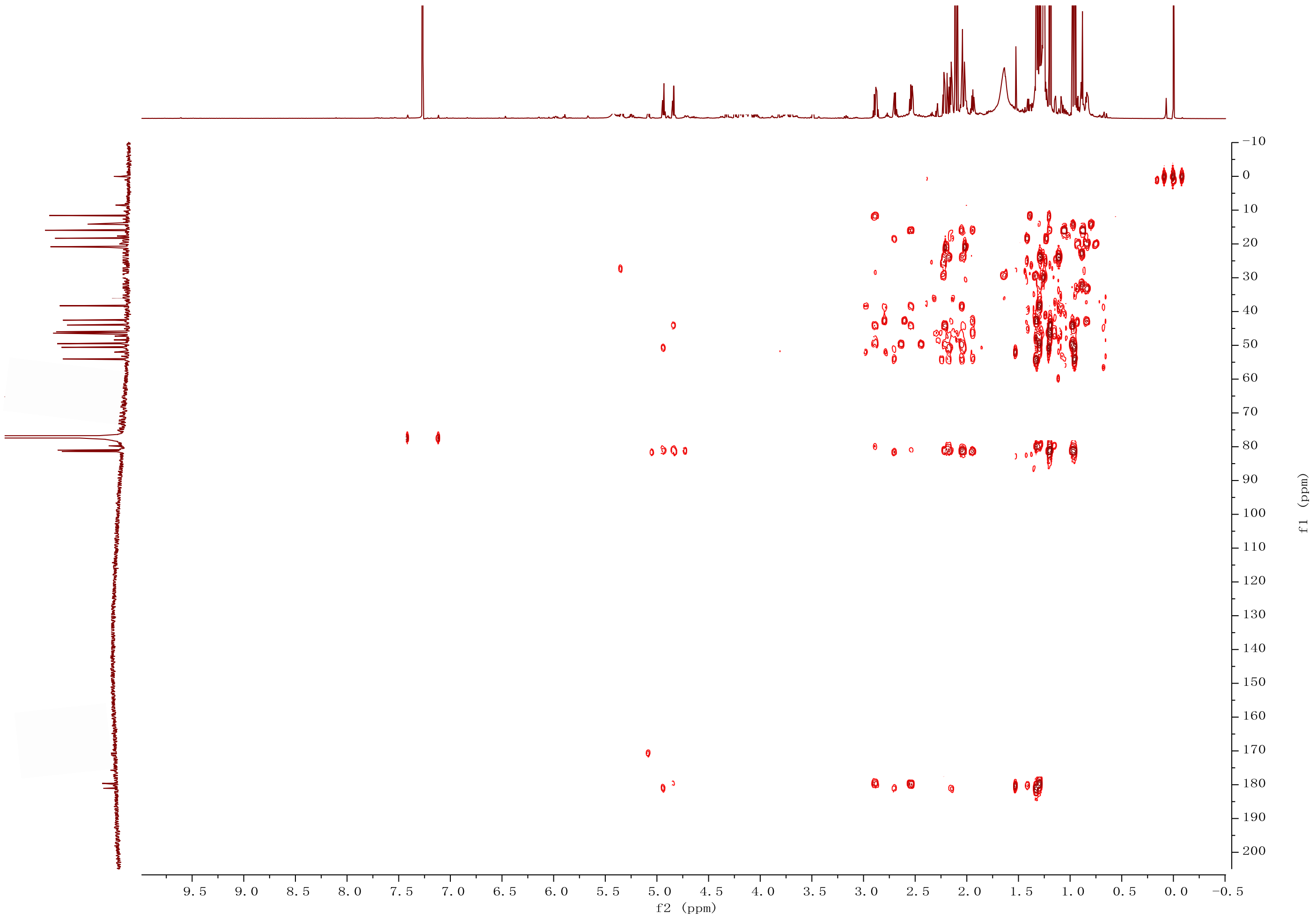

Figure S5. HMBC spectrum of the new compound **1**

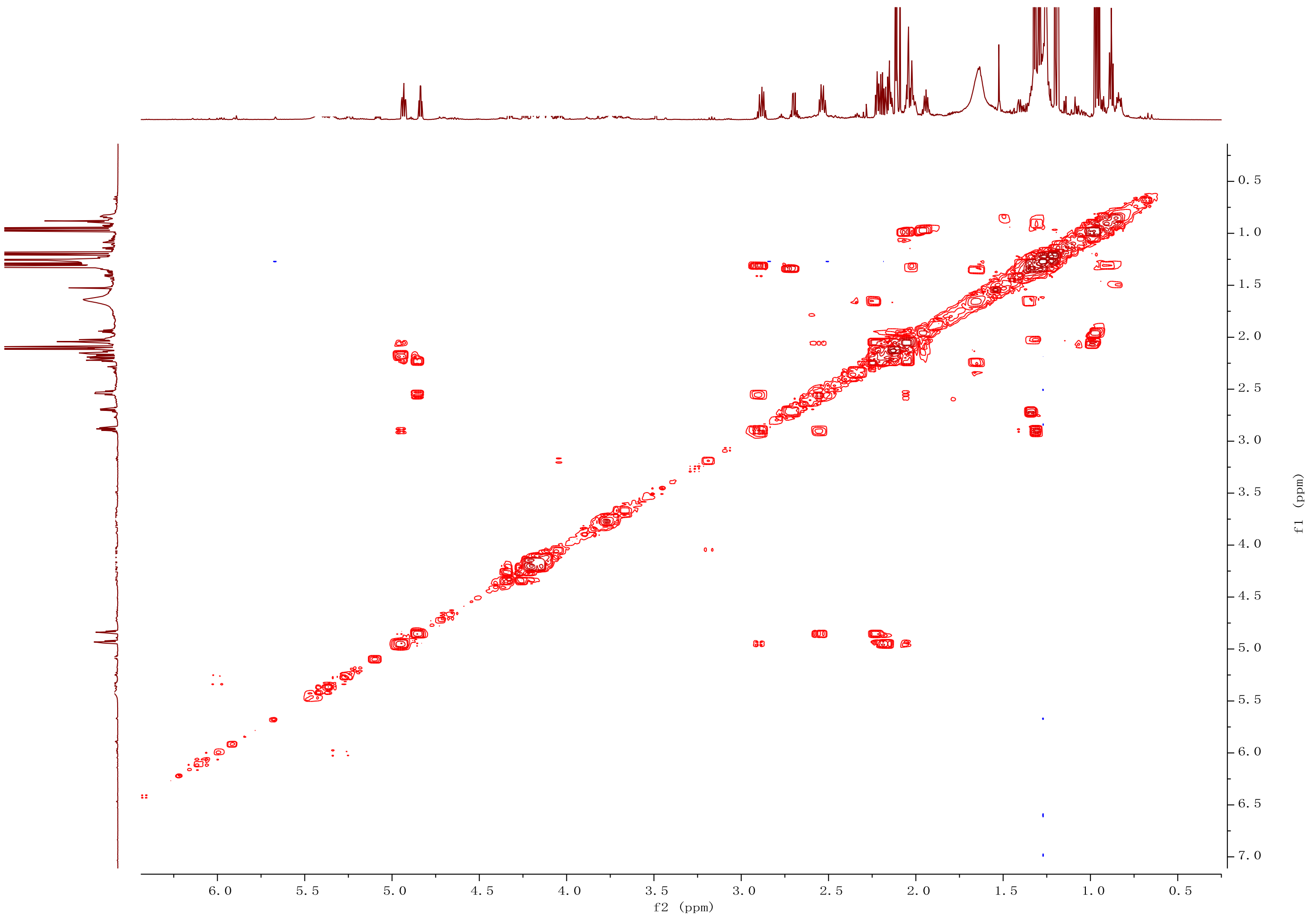

Figure S6. COSY spectrum of the new compound **1**

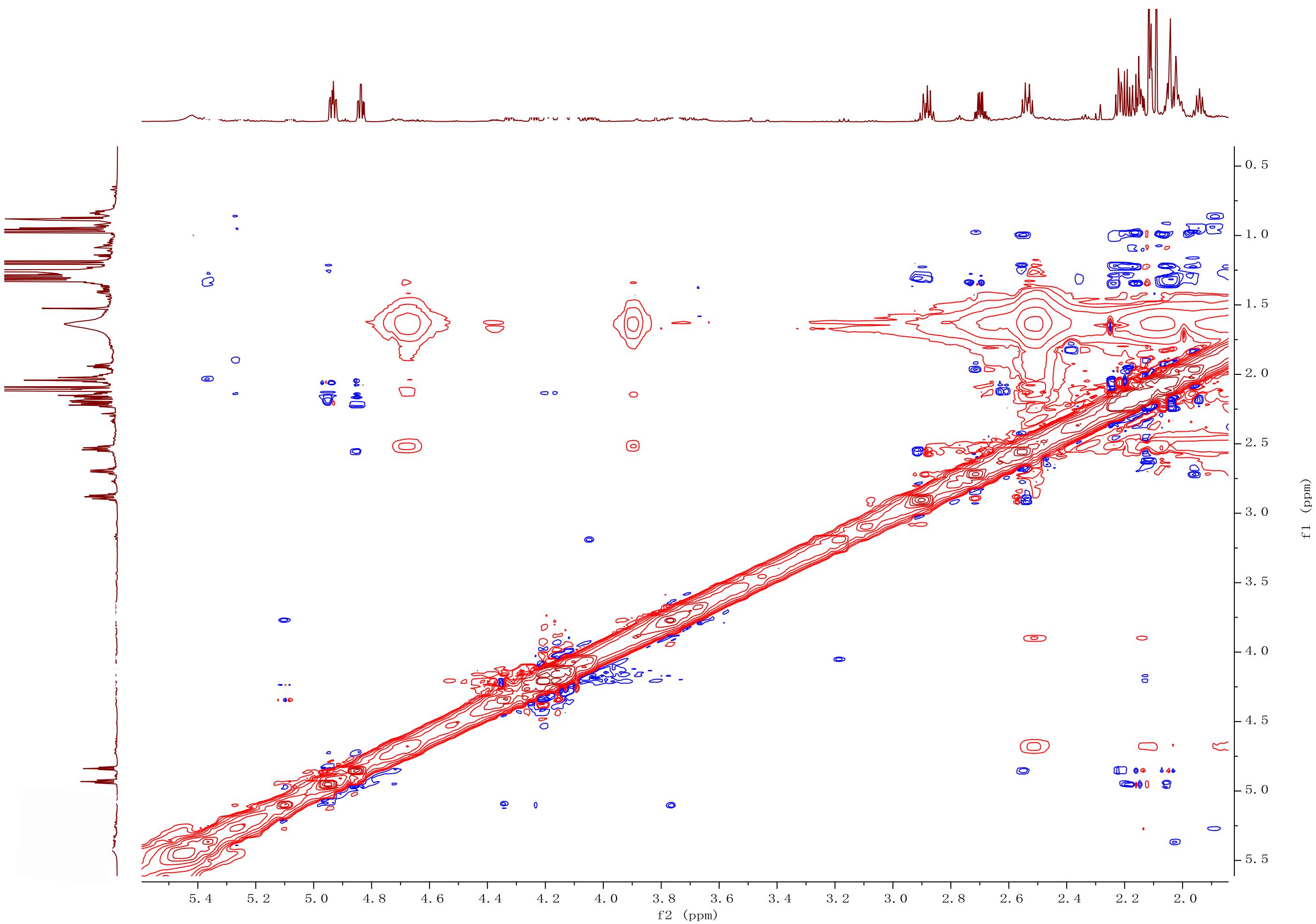

Figure S7. NOESY spectrum of the new compound **1**

Qualitative Analysis Report

**Data Filename**	L13-3-NEG.d	**Sample Name**	Sample11
**Sample Type**	Sample	**Position**	P1-B2
**Instrument Name**	Instrument 1	**User Name**	
**Acq Method**	default-20200902-neg.m	**Acquired Time**	7/1/2021 12:38:29 PM
**IRM Calibration Status**	Some Ions Missed	**DA Method**	analysis.m
**Comment**			

Sample Group **Info.**

User Spectra

Fragmentor Voltage	Collision Energy	Ionization Mode
75	0	ESI

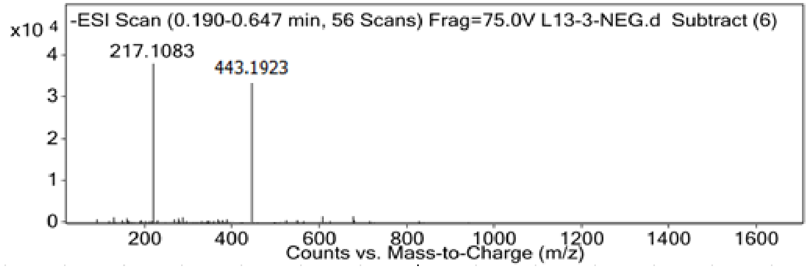

Figure S8. HRESI-MS spectrum of the new compound **2**

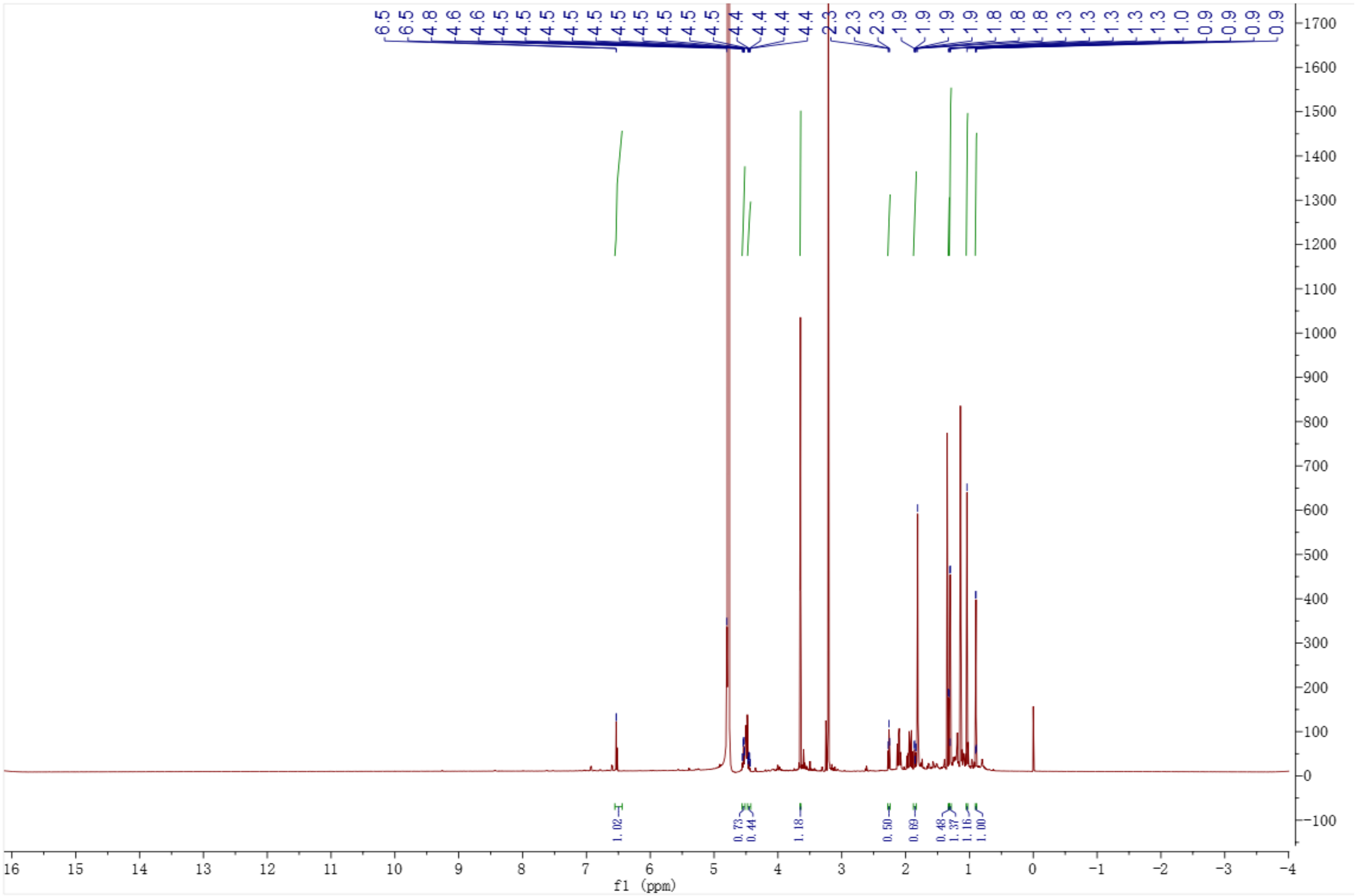

Figure S9. ^{1}H NMR (600 MHz, CD_3OD) spectrum of the new compound **2**

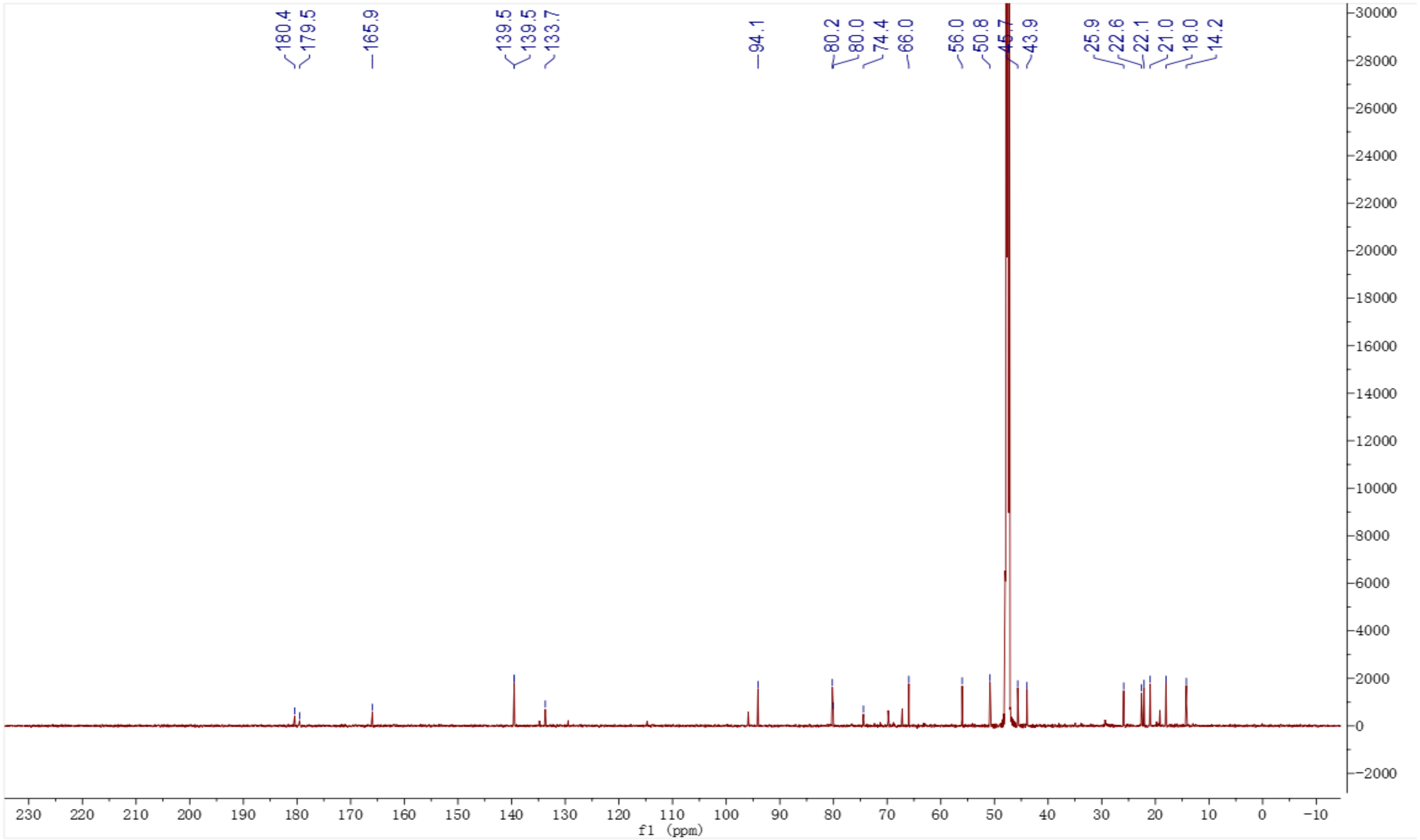

Figure S10. ^{13}C NMR (150 MHz, CD_3OD) spectrum of the new compound **2**

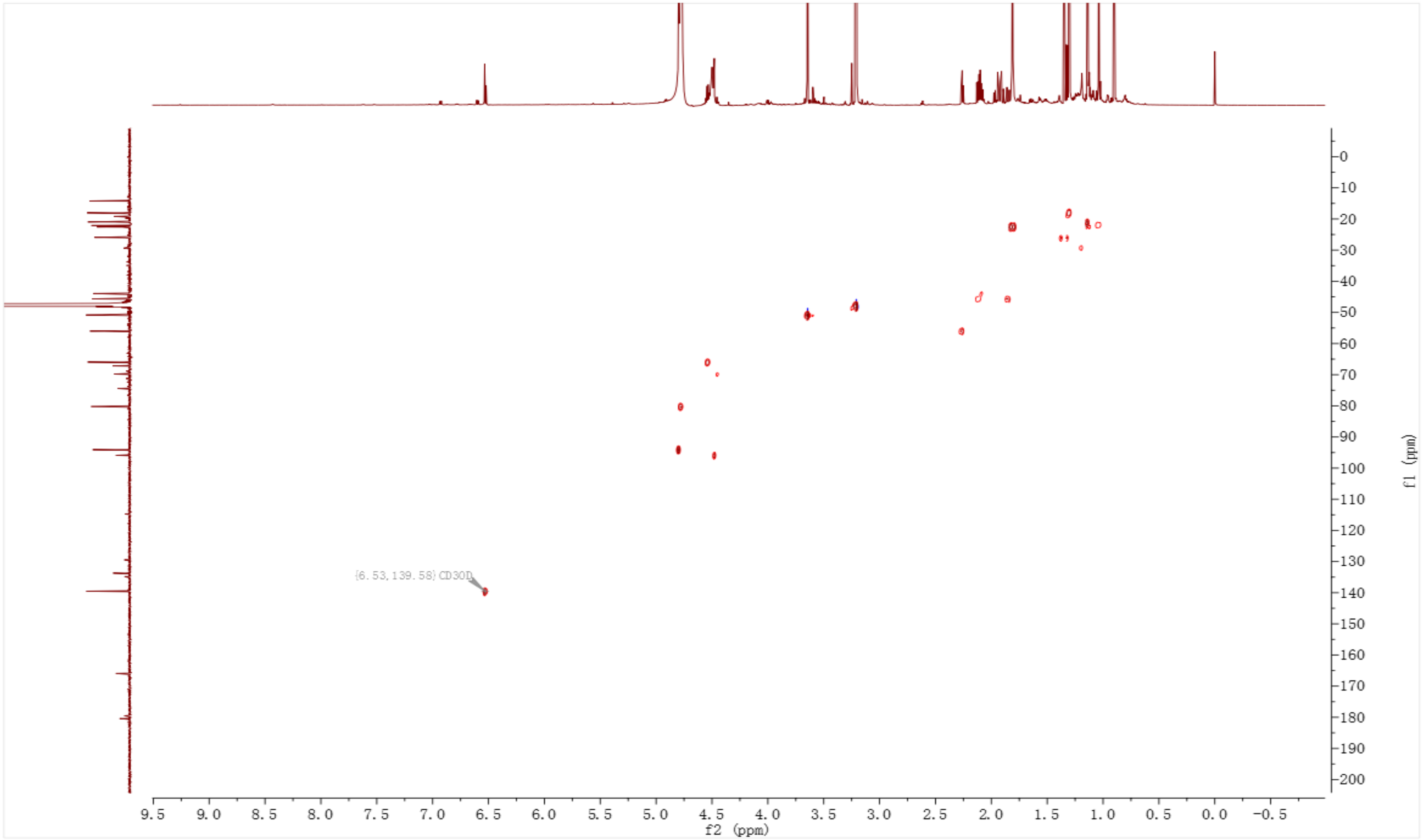

Figure S11. HSQC spectrum of the new compound **2**

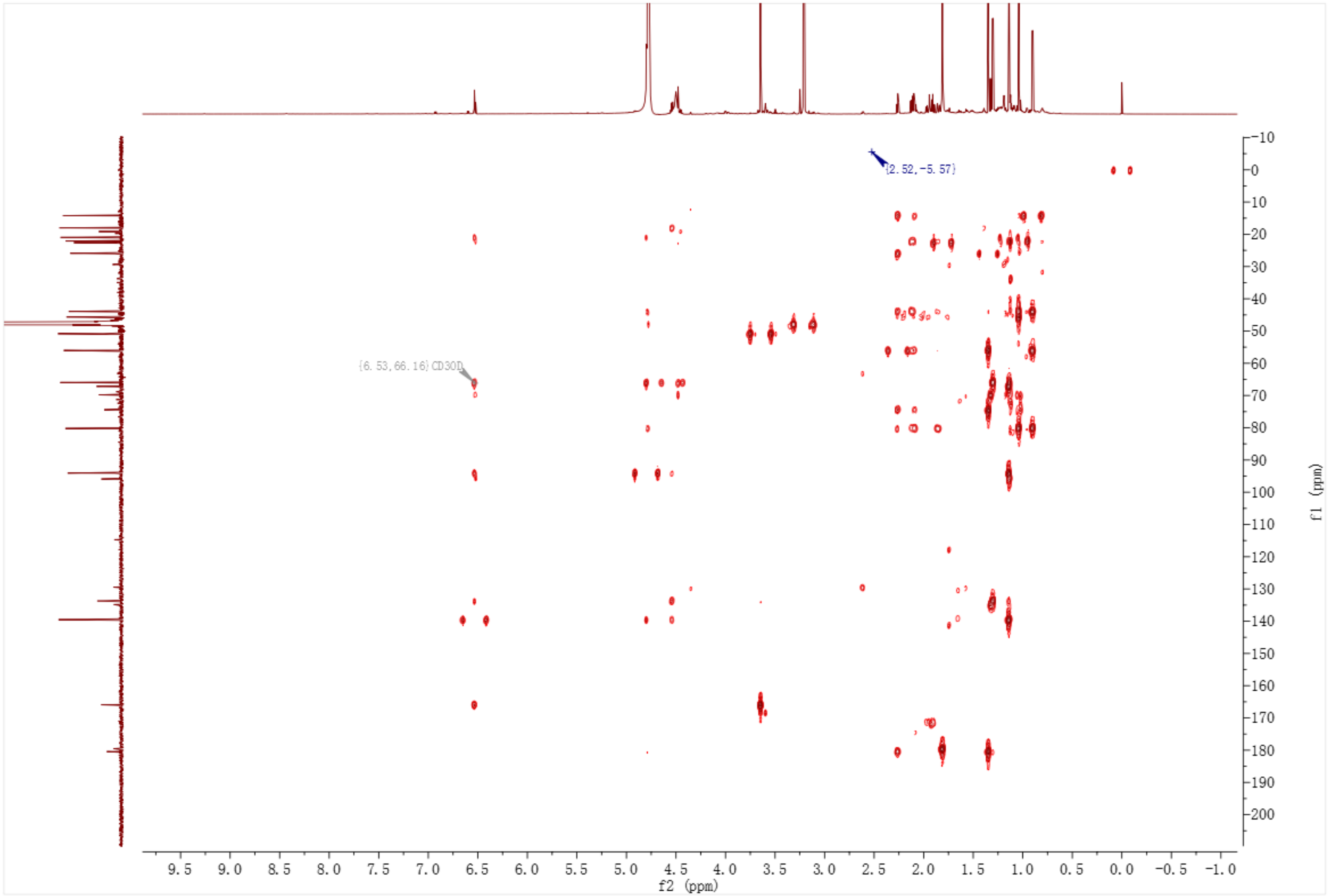

Figure S12. HMBC spectrum of the new compound **2**

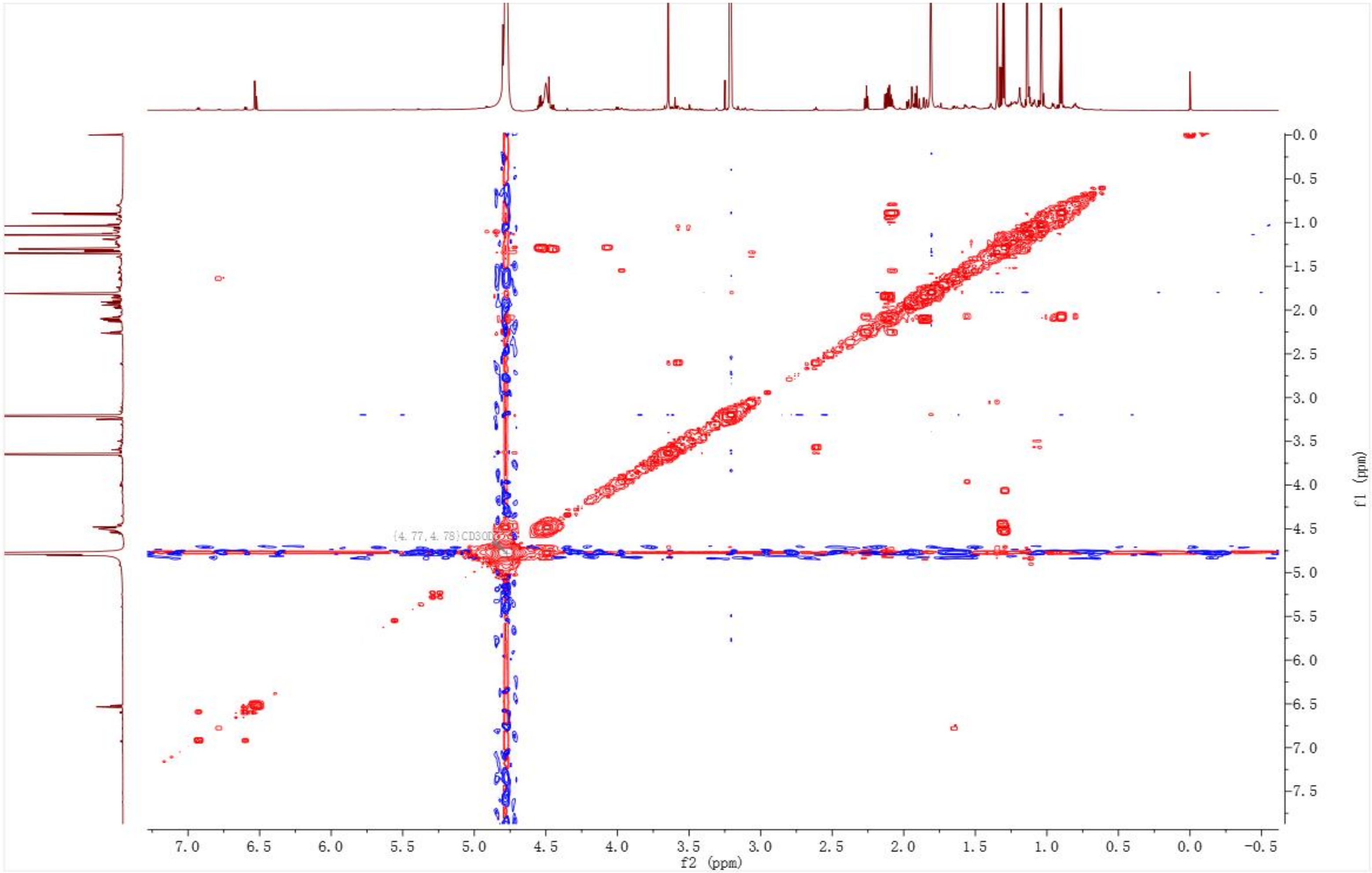

Figure S13. COSY spectrum of the new compound **2**

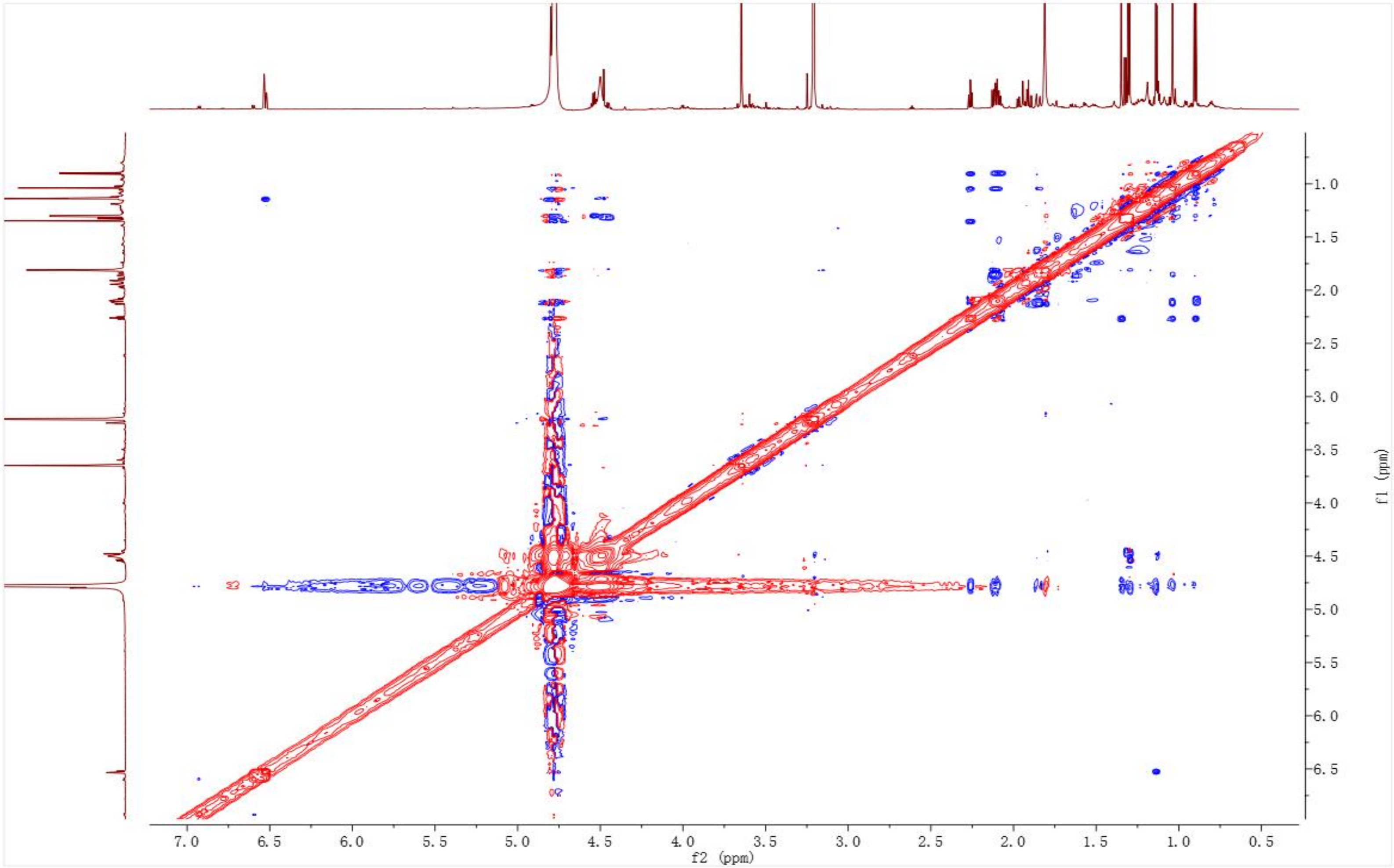

Figure S14. NOESY spectrum of the new compound **2**

Qualitative Analysis Report

**Data Filename**	J-12-POS.d	**Sample Name**	Sample10
**Sample Type**	Sample	**Position**	P1-B1
**Instrument Name**	Instrument 1	**User Name**	
**Acq Method**	default-20200902-pos.m	**Acquired Time**	7/1/2021 9:49:07 AM
**IRM Calibration Status**		**DA Method**	analysis.m
**Comment**			

Sample Group **Info.**

User Spectra

Fragmentor Voltage	Collision Energy	Ionization Mode
75	0	ESI

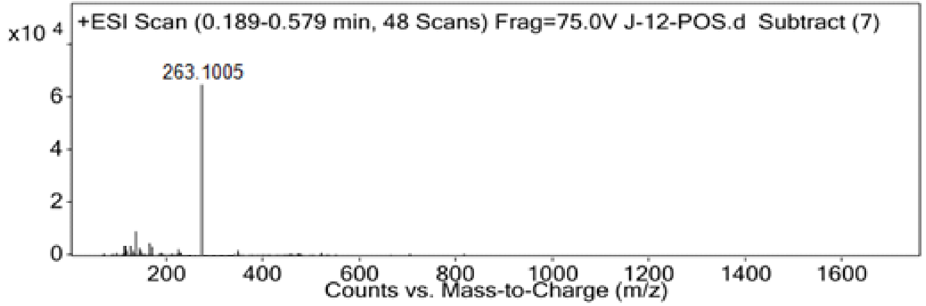

Figure S15. HRESI-MS spectrum of the new compound **3**

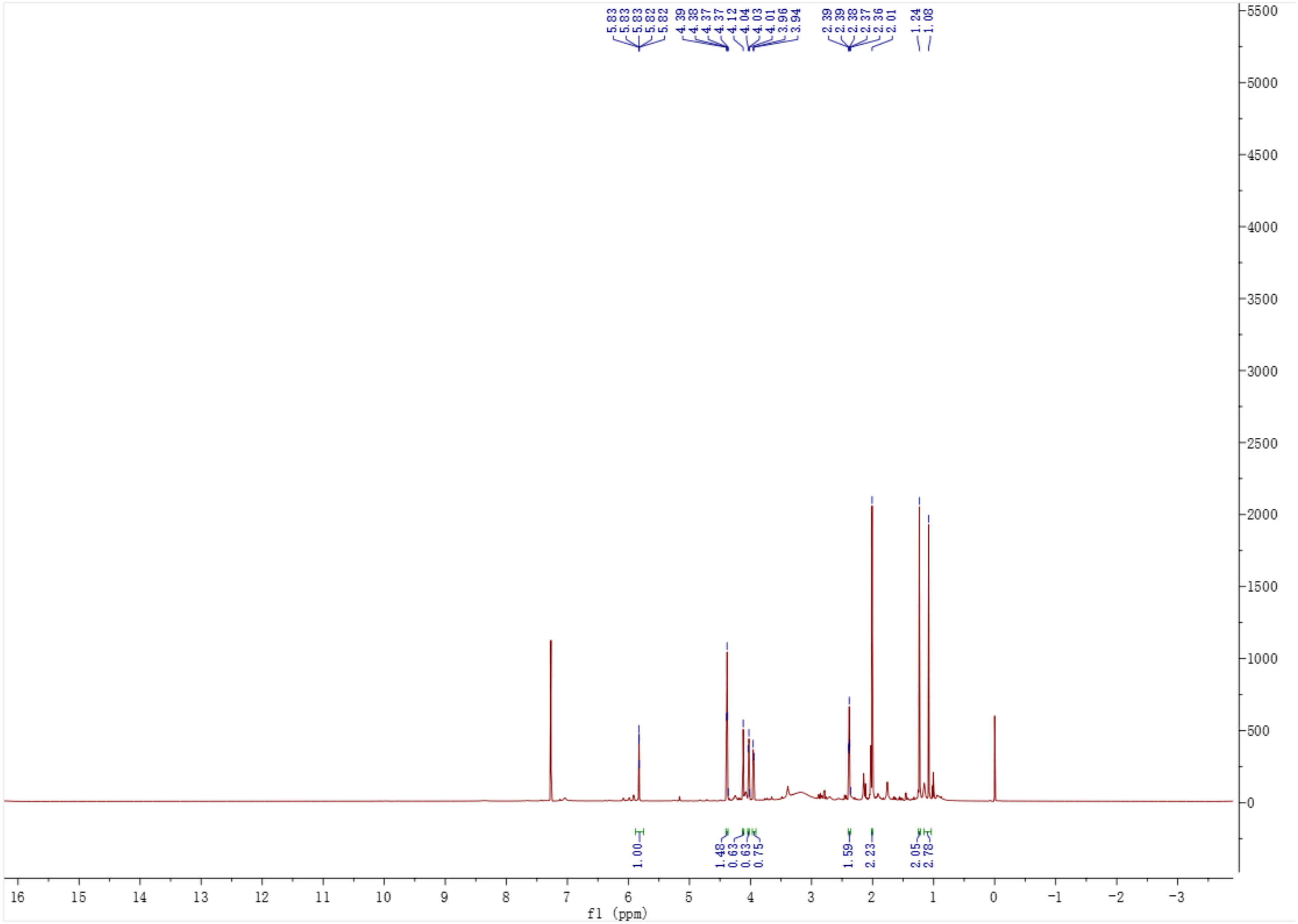

Figure S16. ^{1}H NMR (600 MHz, $CDCl_3$) spectrum of the new compound **3**

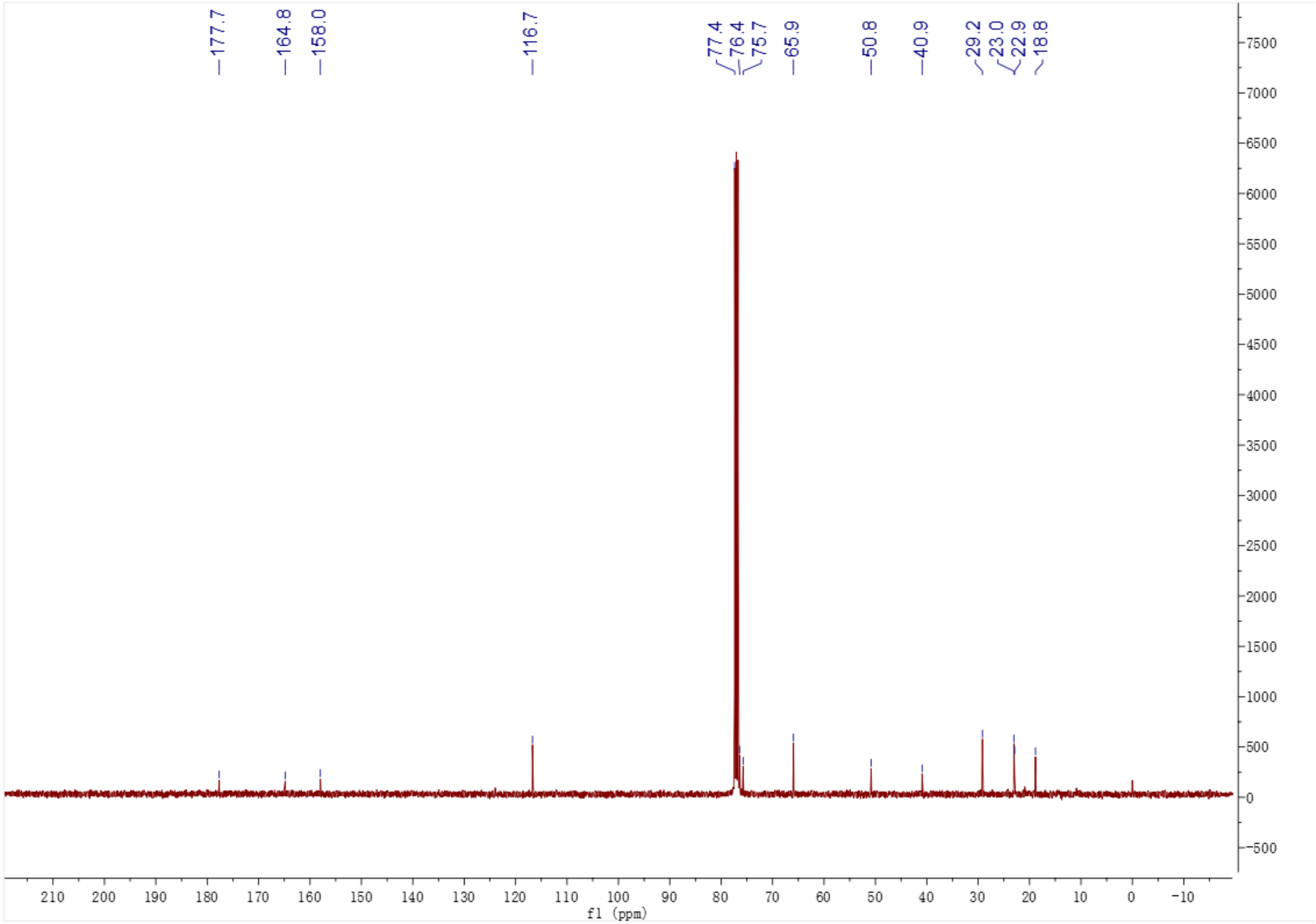

Fiure S17. ^{13}C NMR (150 MHz, $CDCl_3$) spectrum of the new compound **3**

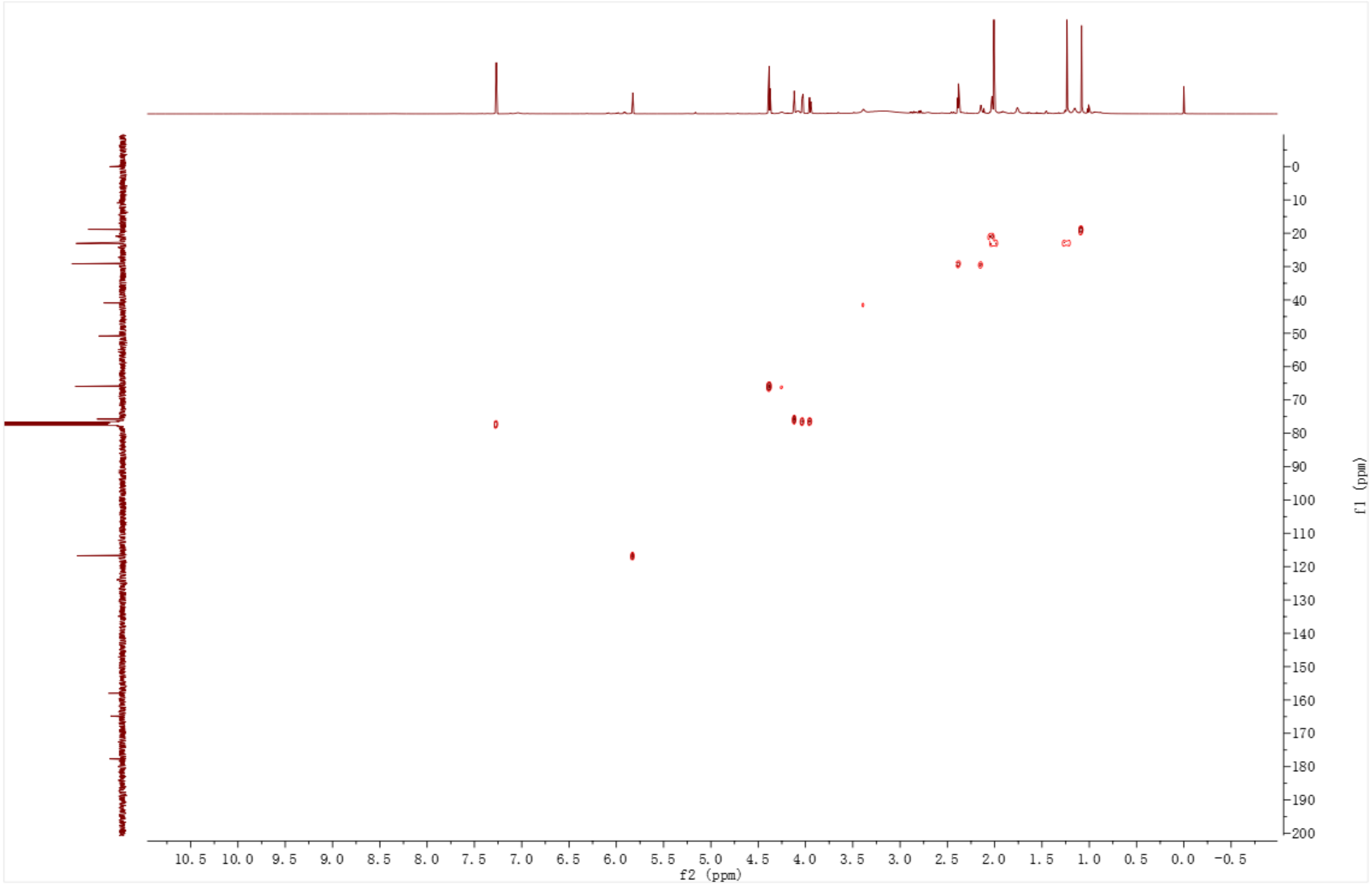

Figure S18. HSQC spectrum of the new compound **3**

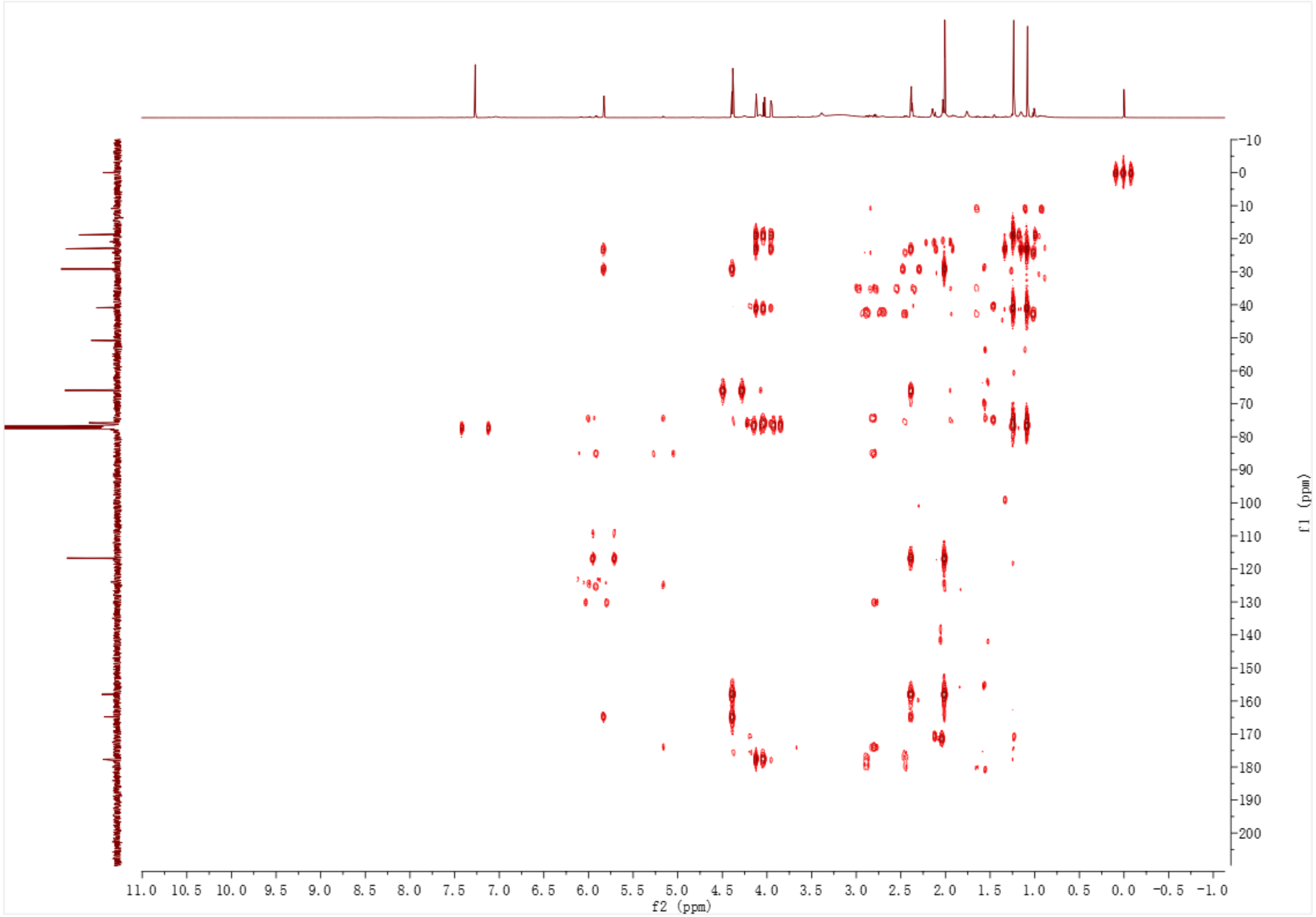

Figure S19. HMBC spectrum of the new compound **3**

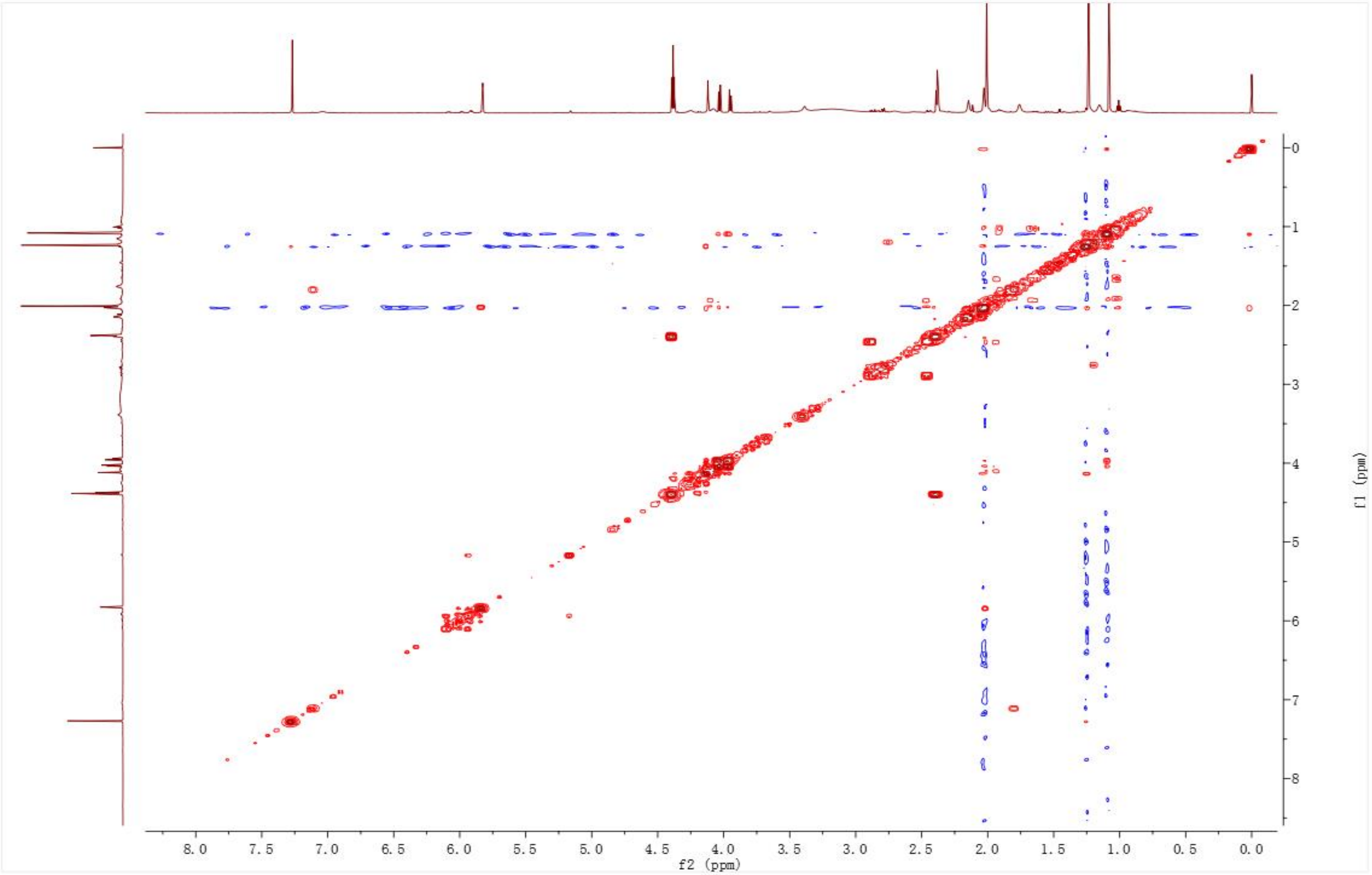

Figure S20. COSY spectrum of the new compound **3**

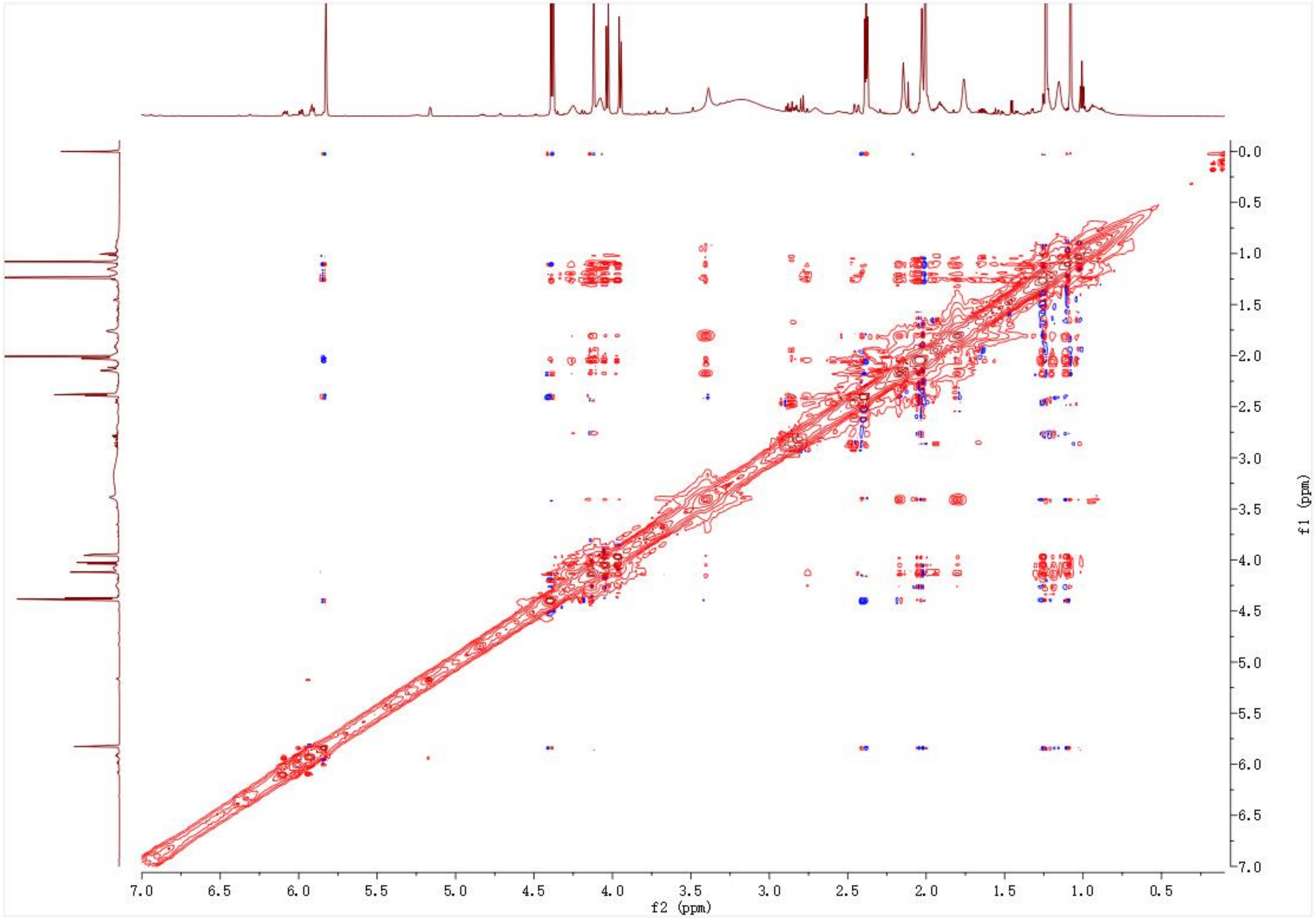

Figure S21. NOESY spectrum of the new compound **3**

Qualitative Analysis Report

**Data Filename**	NEU-105-POS.d Sample	**Sample Name**	Sample13
**Sample Type**	Instrument 1	**Position**	P1-B3
**Instrument Name**	default-20200902-pos.	**User Name**	
**Acq Method**		**Acquired Time**	7/1/2021 13:38:20
**IRM Calibration Status**		**DA Method**	analysis.m
**Comment**			

User Spectra

Fragmentor Voltage	Collision Energy	Ionization Mode
75	0	ESI

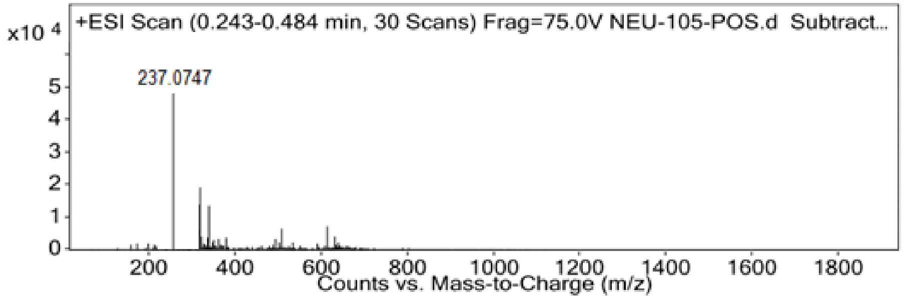

Figure S22. HRESI-MS spectrum of the new compound **4**

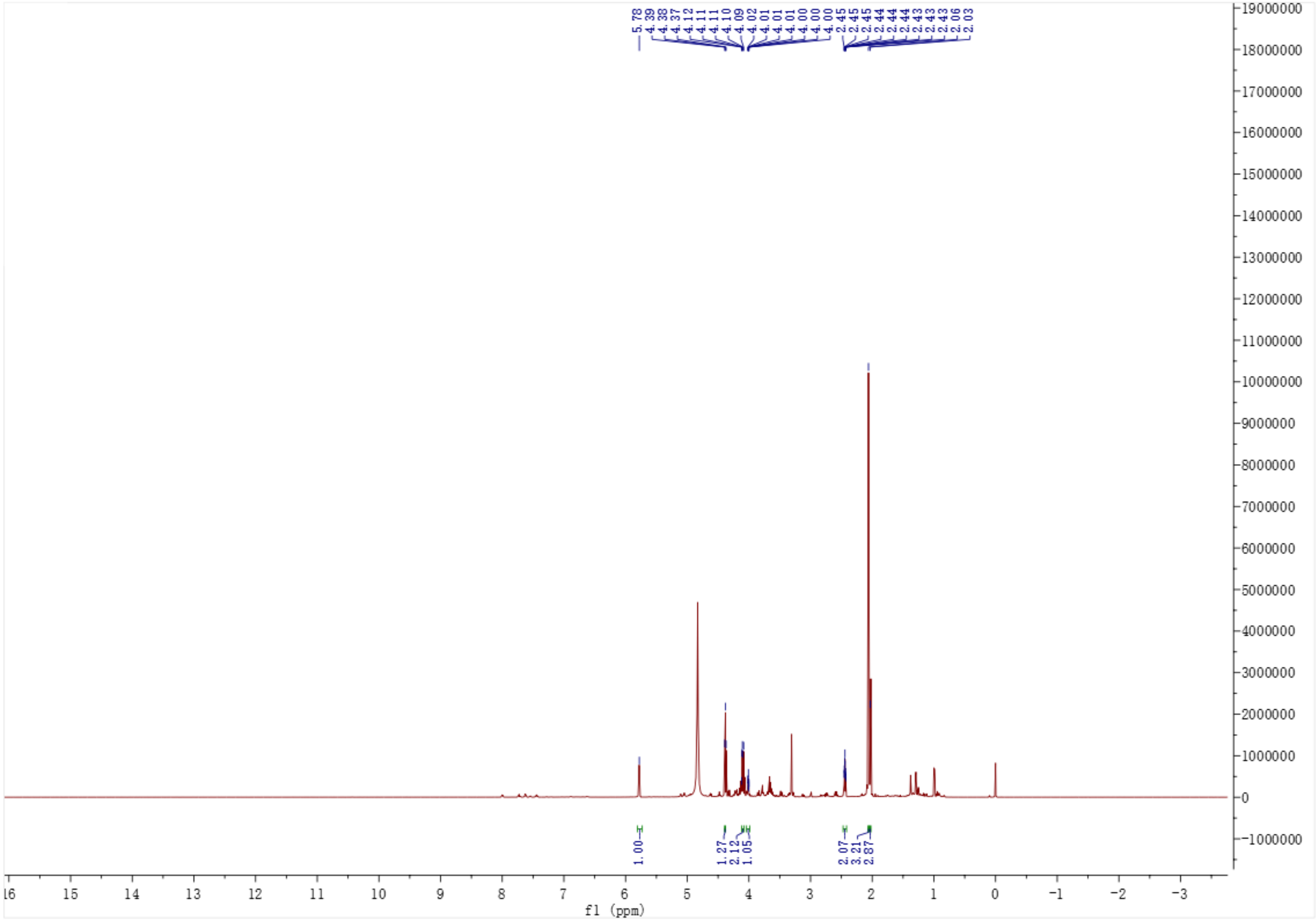

Figure S23. ^{1}H NMR (600 MHz, CD_3OD) spectrum of the new compound **4**

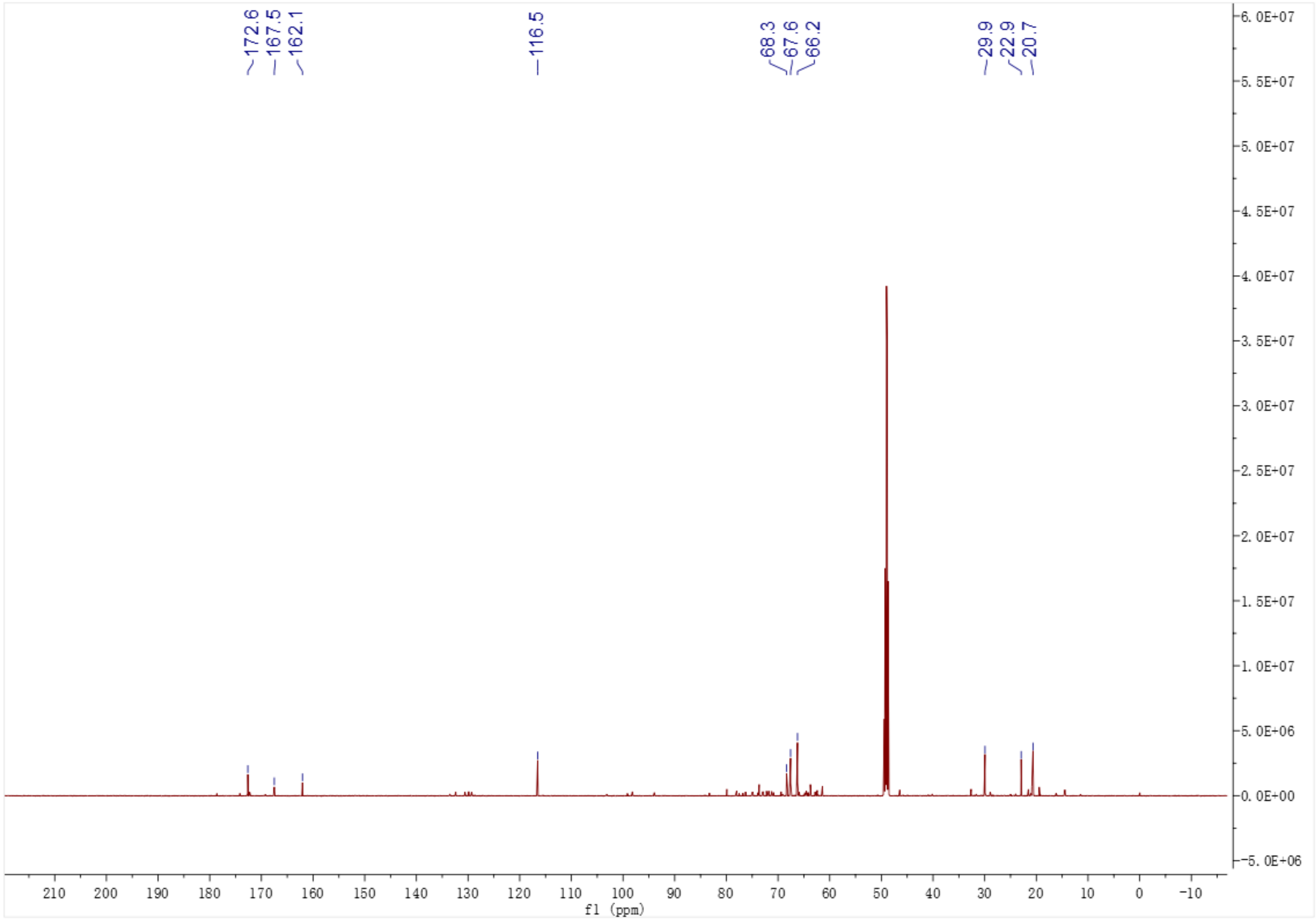

Figure S24. ^{13}C NMR (150 MHz, CD_3OD) spectrum of the new compound **4**

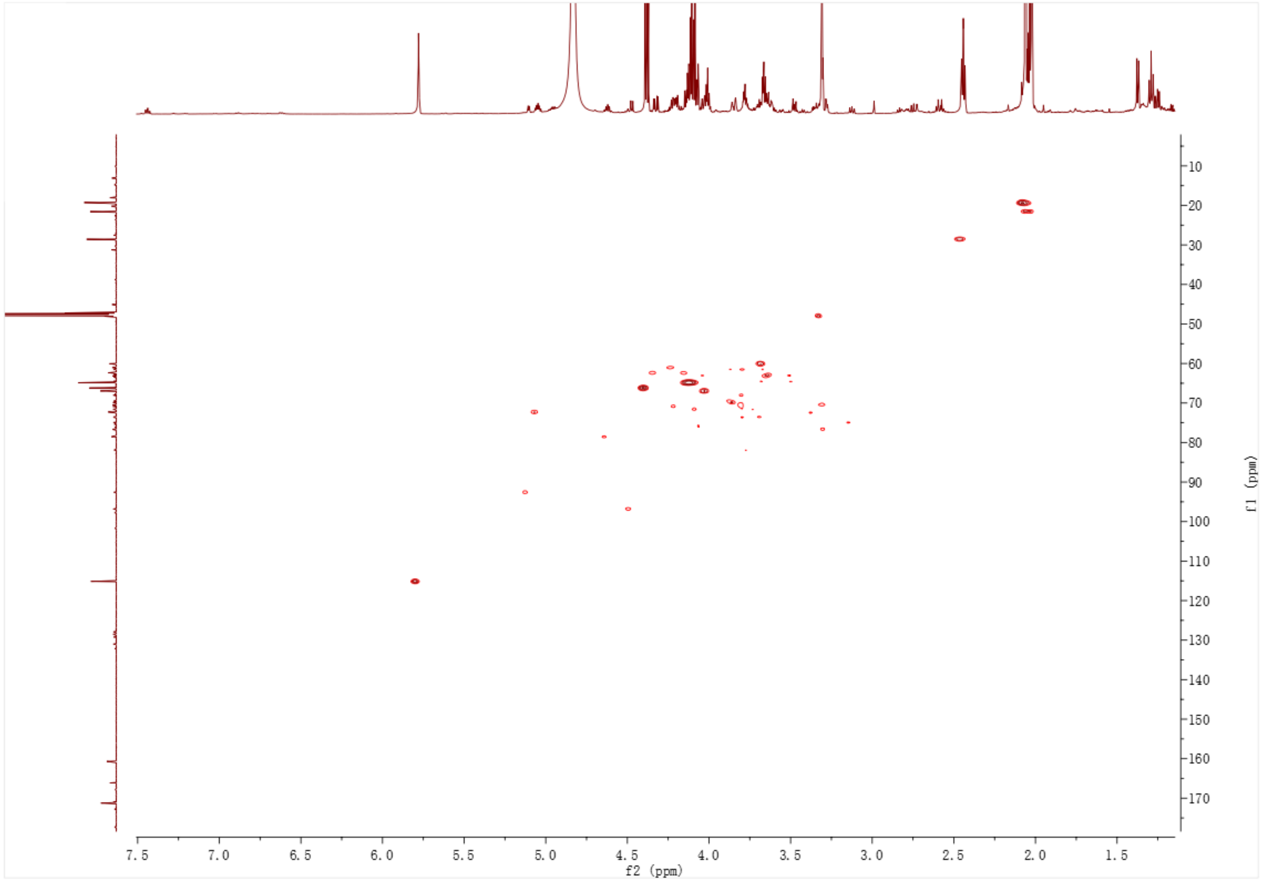

Figure S25. HSQC spectrum of the new compound **4**

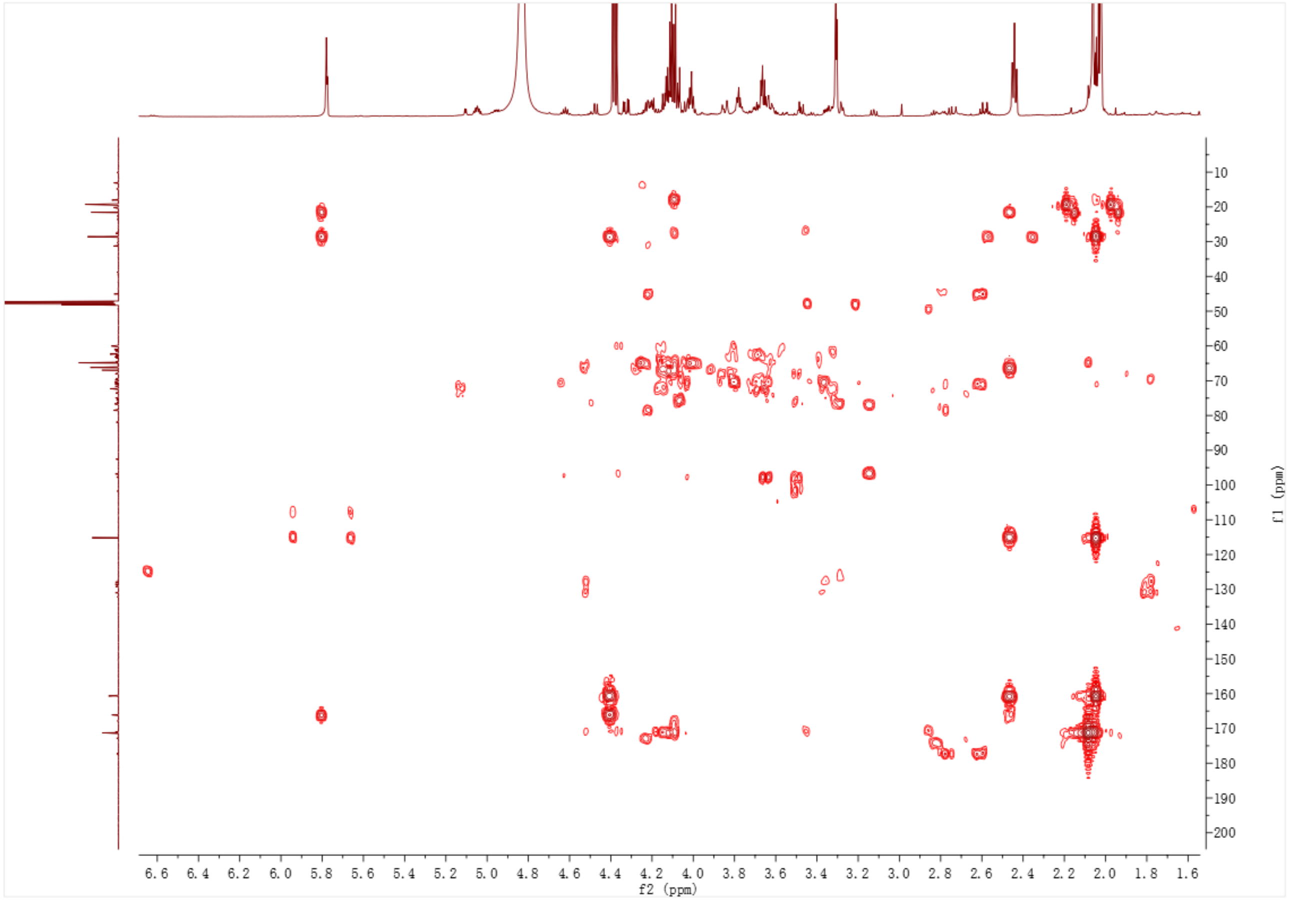

Figure S26. HMBC spectrum of the new compound **4**

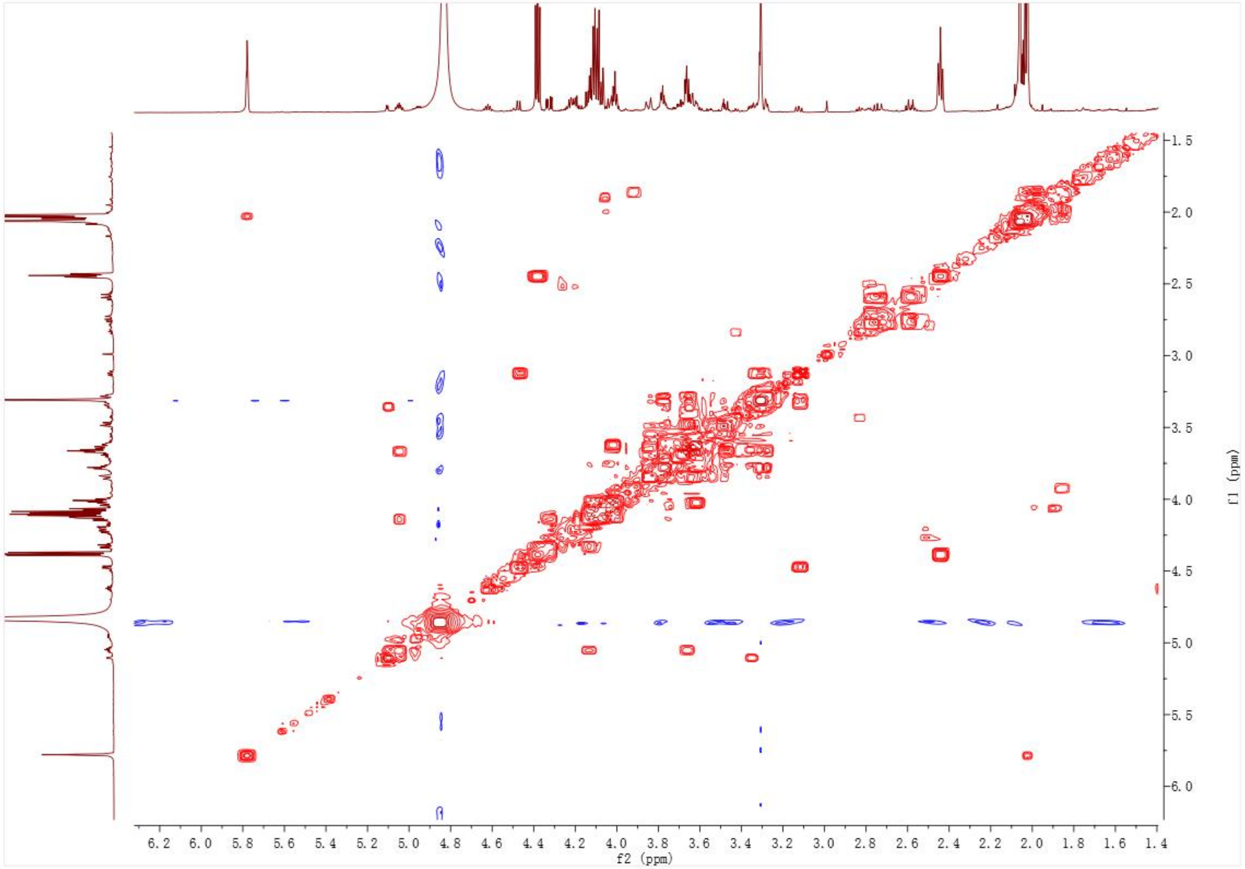

Figure S27. COSY spectrum of the new compound **4**

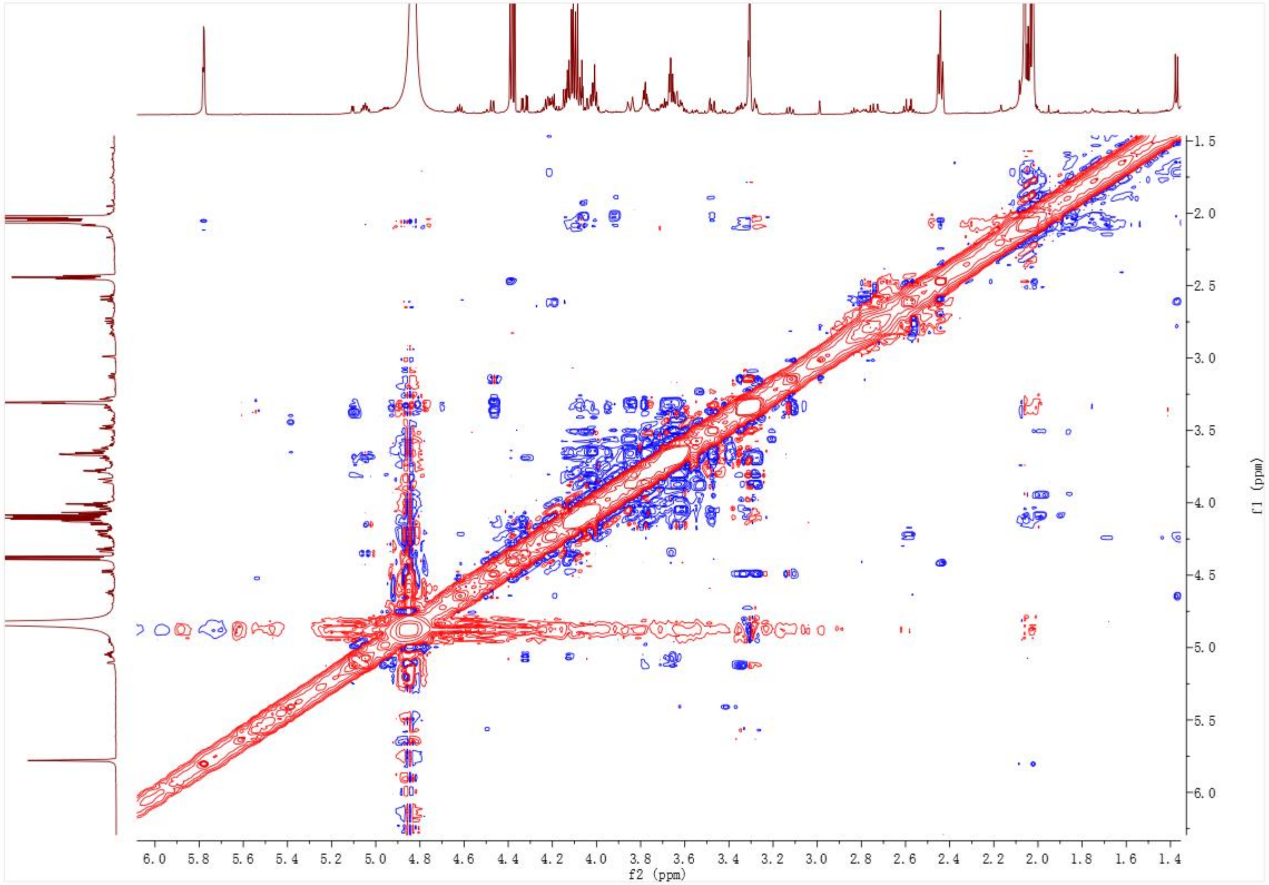

Figure S28. NOESY spectrum of the new compound **4**

Qualitative Analysis Report

**Data Filename**	OMP451-NEG.d	**Sample Name**	Sample13
**Sample Type**	Sample	**Position**	P1-B4
**Instrument Name**	Instrument 1	**User Name**	
**Acq Method**	default-20200902-neg.m	**Acquired Time**	7/1/2021 12:42:16 PM
**IRM Calibration Status**	Some Ions Missed	**DA Method**	analysis.m
**Comment**			

Sample Group **Info.**

User Spectra

Fragmentor Voltage	Collision Energy	Ionization Mode
75	0	ESI

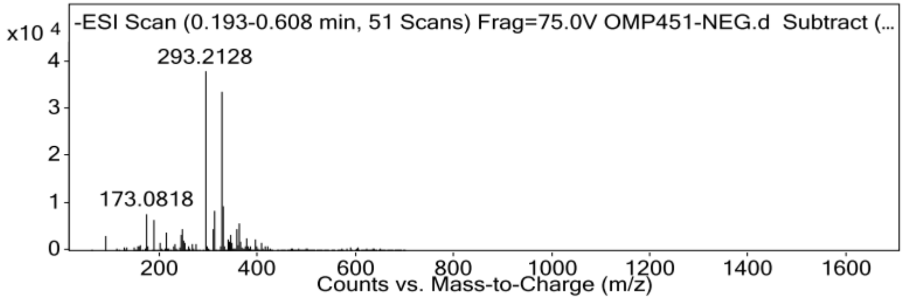

Figure S29. HRESI-MS spectrum of the new compound **5**

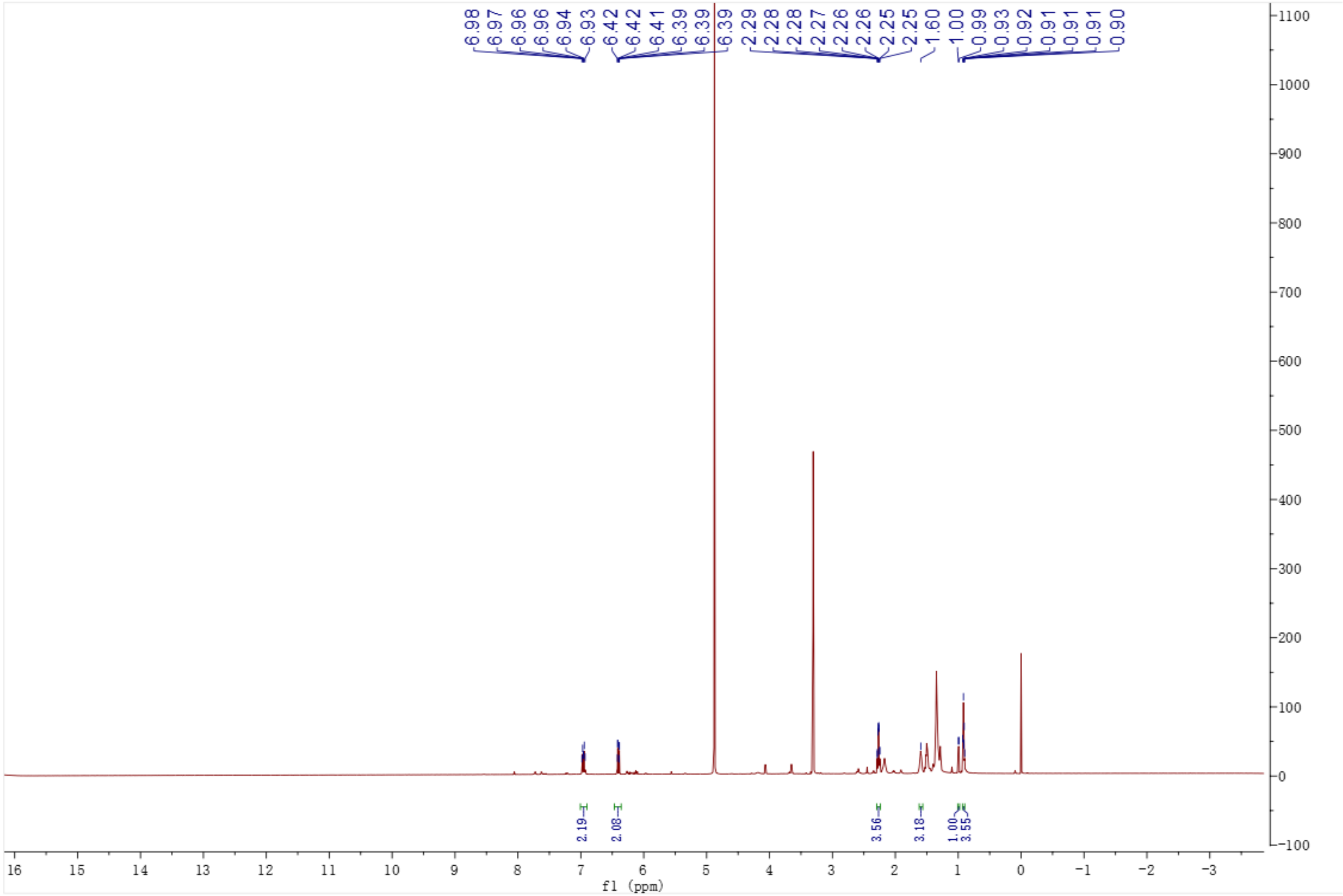

Figure S30. ^{1}H NMR (600 MHz, CD_3OD) spectrum of the new compound **5**

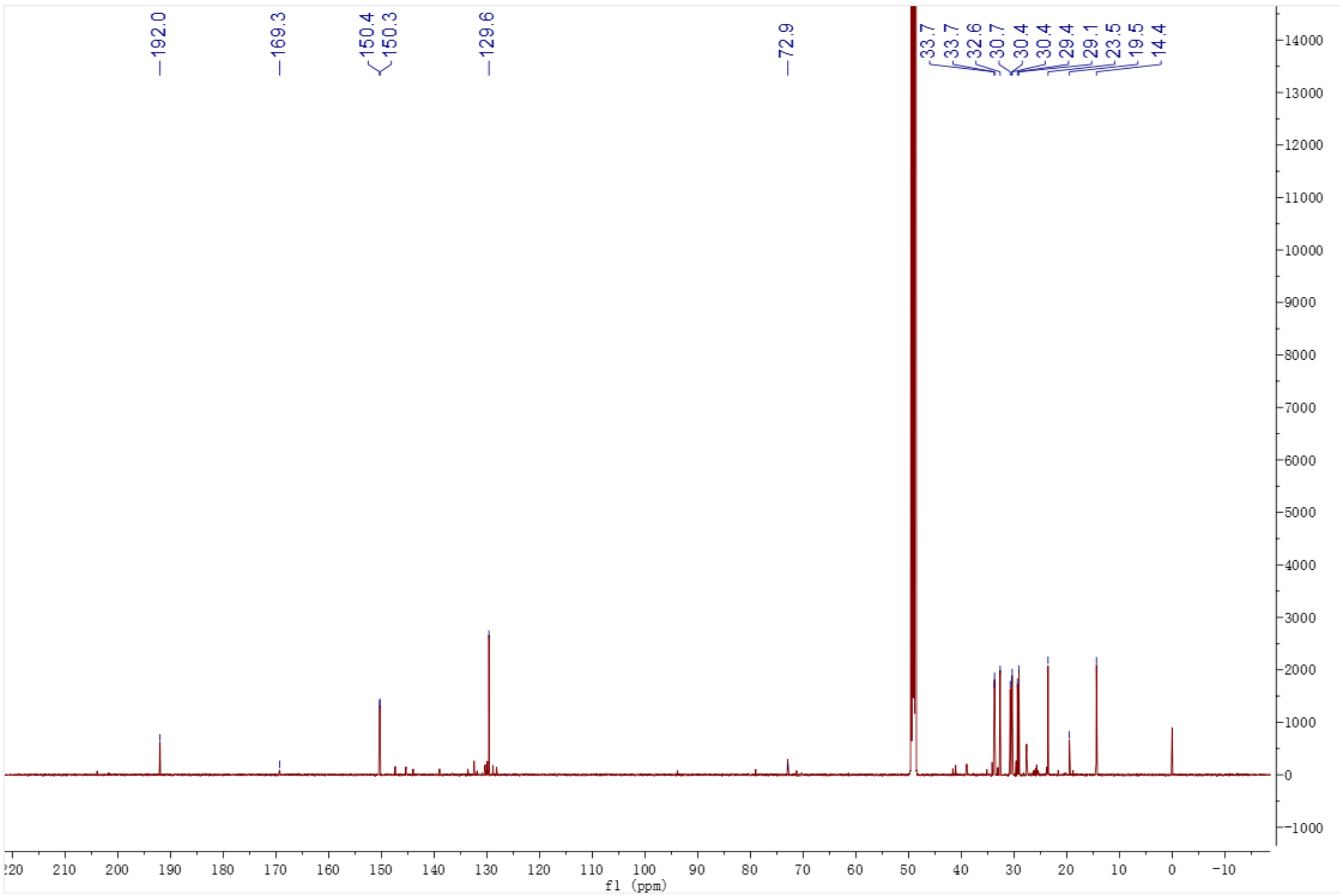

Figure S31. ^{13}C NMR (150 MHz, CD_3OD) spectrum of the new compound **5**

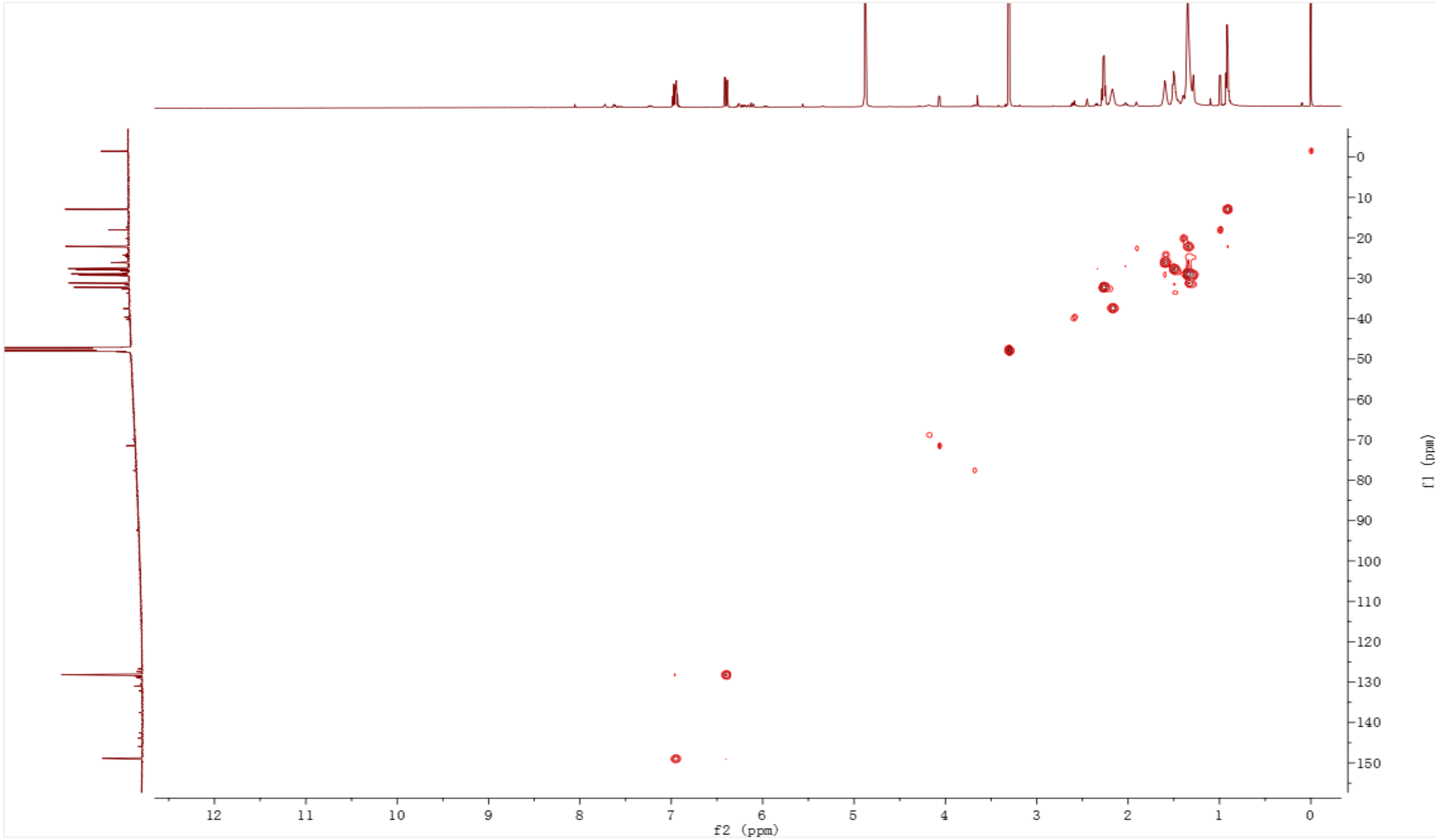

Figure S32. HSQC spectrum of the new compound **5**

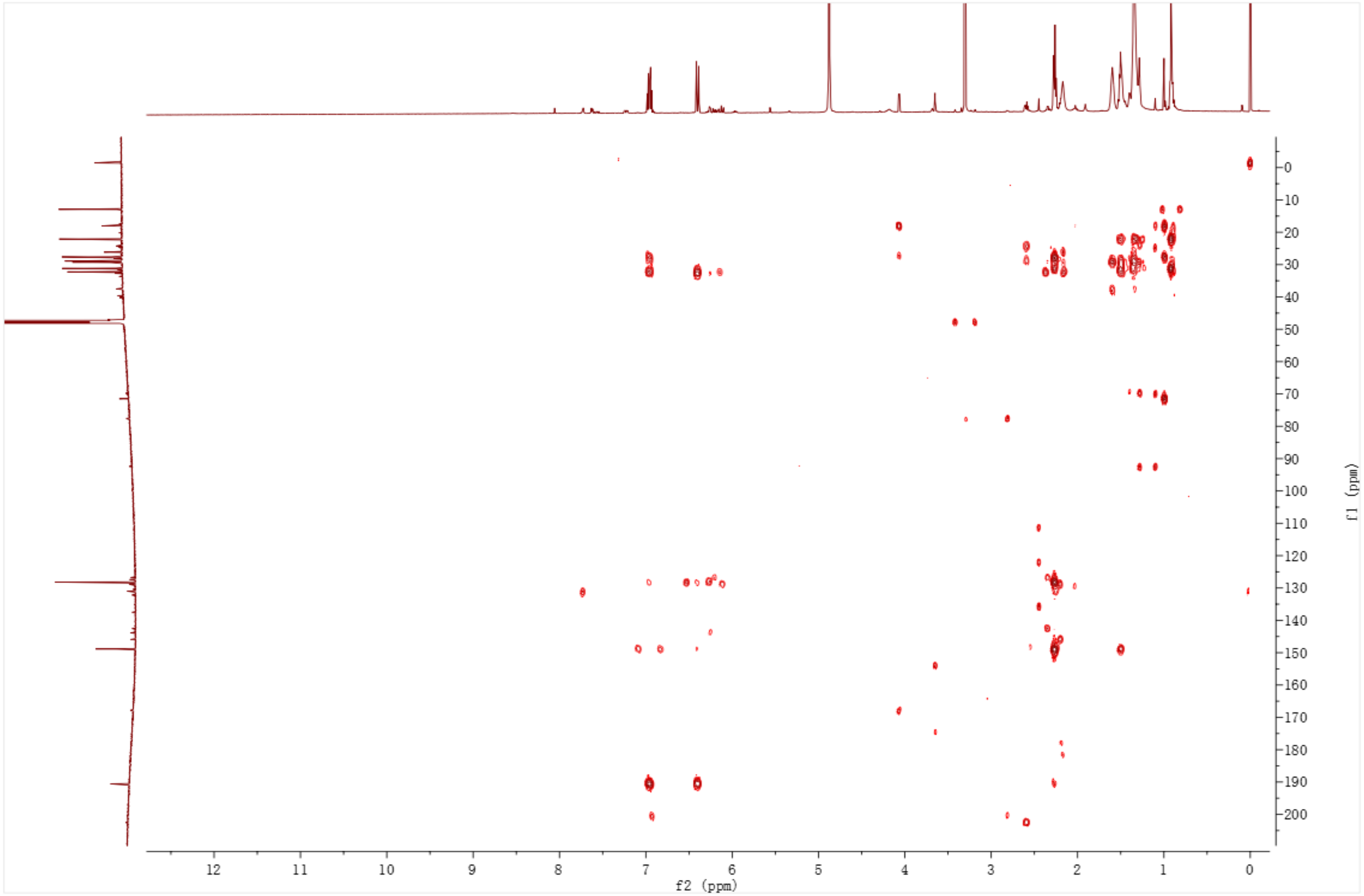

Figure S33. HMBC spectrum of the new compound **5**

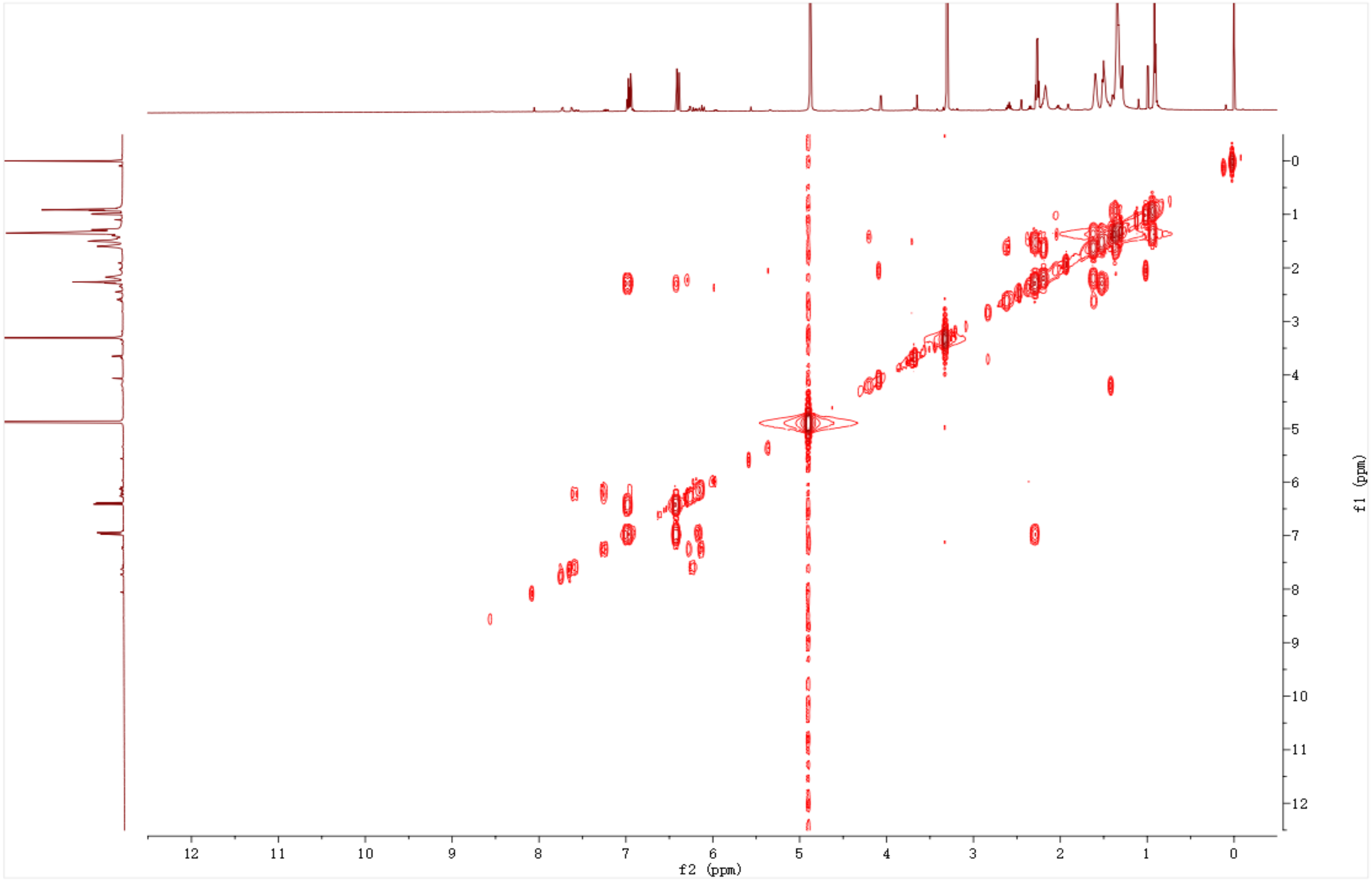

Figure S34. COSY spectrum of the new compound **5**

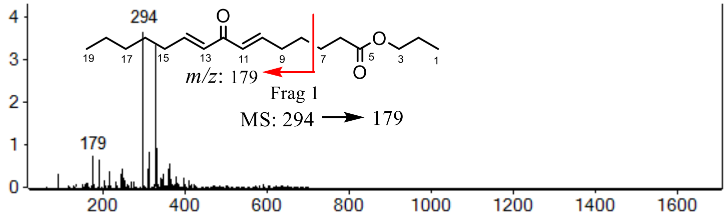

Figure S35. ESI-MS spectrum of compound **5**.

S36. Computational details for compound 1-4 (ECD)

The calculations of new compounds **1-4** were performed by using the density functional theory (DFT) as carried out in the Gaussian 09. Conformation search were performed with MMFF94S force fields using MacroModel software. All these conformers were further optimized by the density functional theory method at the B3LYP/6-31G(d) level (Boltzmann distribution over 5%, the relative energy within 6 kcal/mol). The ECD were calculated using density functional theory (TDDFT) at B3LYP/6-31+G(d) level. The ECD spectra of different conformers were generated using the program SpecDis by applying a Gaussian band shape with a 0.30 eV width, according to the Boltzmann-calculated contribution after UV correction.

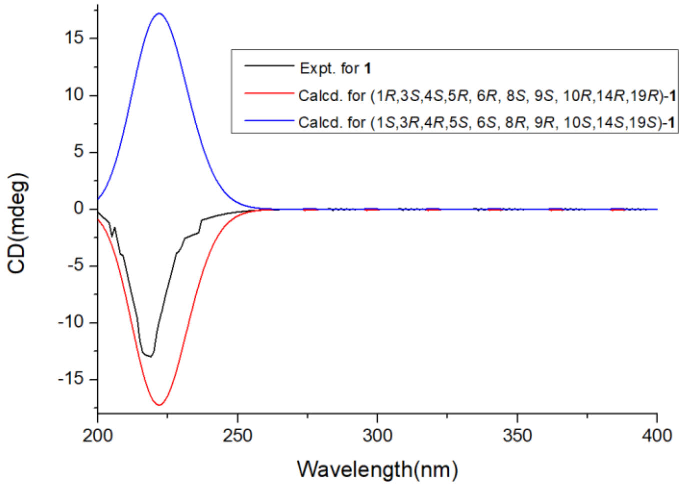

Figure S37. Comparison of the calculated ECD spectra for **1** with the experimental spectrum of **1**

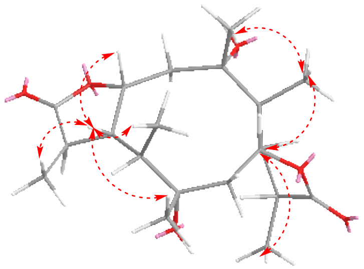

1

1*R*, 3*S*, 4*S*, 5*R*, 6*R*, 8*S*, 9*S*, 10*R*, 14*R*, 19*R*-M-**1**.

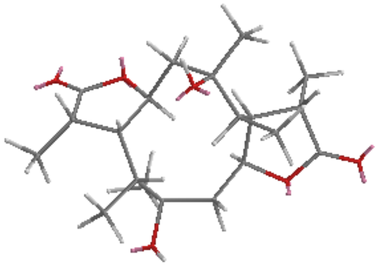

1*S*, 3*R*, 4*R*, 5*S*, 6*S*, 8*R*, 9*R*, 10*S*, 14*S*, 19*S*-M-**1**

Figure S38. calculated and experimental ECD spectras of **1** using model.

Table S1. Energy analysis for conformers of **1** at B3LYP/6-31G(d) level

**Conformer**	**Energy**（hartree）	**Ratio**（%）
1	-864.14735	1.71
2	-864.14747	1.27
4	-864.14833	2.85
6	-864.14831	3.09
10	-864.13144	89.09
12	-864.14767	1.41
16	-864.11758	1.43
20	-864.11825	3.98
36	-864.11793	2.29
40	-864.11826	3.27
66	-864.12736	1.26
70	-864.12871	4.24
84	-864.12812	2.81
89	-864.12738	1.29
93	-864.12614	1.38

96	-864.12813	3.26
98	-864.12946	4.31

Table S2. Energy analysis for conformers of **2** at B3LYP/6-31G(d) level

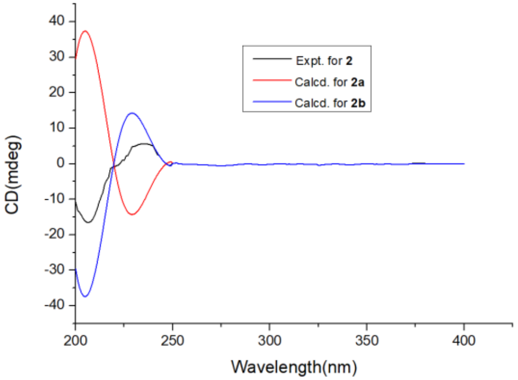

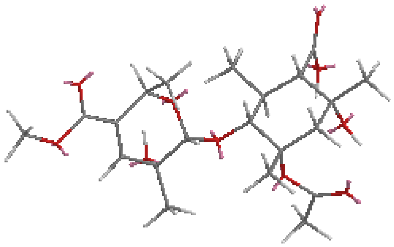

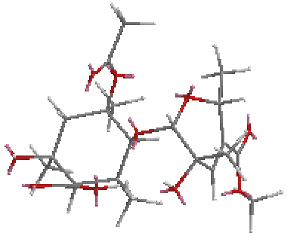

Compound 2 Model-1 model-2

Conformer	Energy（hartree）	Ratio（%）
2	-734.6934336	7.45
3	-734.6952926	2.64
4	-734.6939118	64.45
5	-734.6945719	9.36
8	-734.6941533	6.23
9	-734.6958112	4.16

10	-734.6955858	3.25
12	-734.6957623	4.65
13	-734.6944719	8.01
14	-734.6951045	6.34
15	-734.6941238	2.18
16	-734.6941029	6.01
21	-734.6956123	3.93
22	-734.6952278	3.48
25	-734.6956833	3.61
27	-734.6952934	3.08
29	-734.6959934	4.31
30	-734.6958118	5.18

Table S3. Energy analysis for conformers of **3** at B3LYP/6-31G(d) level

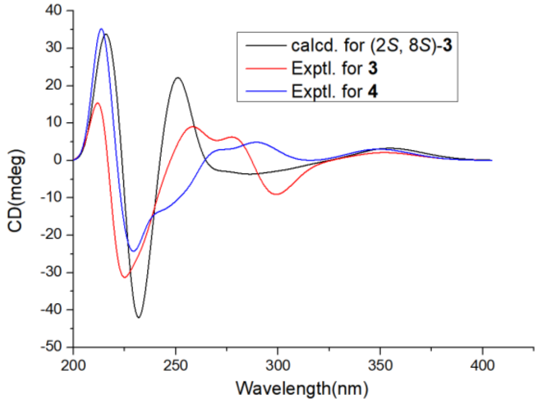

**conformer**	**Energy（hartree）**	**Ratio（%）**
1	-794.6644631	68.52
2	-794.6626493	28.32
4	-794.6620634	2.30
5	-794.6620483	1.57
